# Alpha-Synuclein defects autophagy by impairing SNAP29-mediated autophagosome-lysosome fusion

**DOI:** 10.1038/s41419-021-04138-0

**Published:** 2021-09-17

**Authors:** Qilin Tang, Pan Gao, Thomas Arzberger, Matthias Höllerhage, Jochen Herms, Günter Höglinger, Thomas Koeglsperger

**Affiliations:** 1 Department of Translational Brain Research, German Center for Neurodegenerative Diseases (DZNE), Munich, Germany; 2 Department of Neurology, Ludwig Maximilian University, Munich, Germany; 3 Technical University of Munich, Munich, Germany; 4 Centre of Neuropathology and Prion Research, Ludwig, Maximilian University, Munich, Germany; 5 Department of Psychiatry and Psychotherapy, Ludwig Maximilian University, Munich, Germany; 6 Department of Neurology, Hannover Medical School (MHH), Hannover, Germany; 7 Munich Cluster for Systems Neurology (SyNergy), Munich, Germany; 8 Department of Translational Neurodegeneration, German Center for Neurodegenerative Diseases (DZNE), Munich, Germany

**Keywords:** Cell death in the nervous system, Parkinson's disease

## Abstract

Dopaminergic (DA) cell death in Parkinson’s disease (PD) is associated with the gradual appearance of neuronal protein aggregates termed Lewy bodies (LBs) that are comprised of vesicular membrane structures and dysmorphic organelles in conjunction with the protein alpha-Synuclein (α-Syn). Although the exact mechanism of neuronal aggregate formation and death remains elusive, recent research suggests α-Syn-mediated alterations in the lysosomal degradation of aggregated proteins and organelles – a process termed autophagy. Here, we used a combination of molecular biology and immunochemistry to investigate the effect of α-Syn on autophagy turnover in cultured human DA neurons and in human post-mortem brain tissue. We found α-Syn overexpression to reduce autophagy turnover by compromising the fusion of autophagosomes with lysosomes, thus leading to a decrease in the formation of autolysosomes. In accord with a compensatory increase in the plasma membrane fusion of autophagosomes, α-Syn enhanced the number of extracellular vesicles (EV) and the abundance of autophagy-associated proteins in these EVs. Mechanistically, α-Syn decreased the abundance of the v-SNARE protein SNAP29, a member of the SNARE complex mediating autophagolysosome fusion. In line, SNAP29 knockdown mimicked the effect of α-Syn on autophagy whereas SNAP29 co-expression reversed the α-Syn-induced changes on autophagy turnover and EV release and ameliorated DA neuronal cell death. In accord with our results from cultured neurons, we found a stage-dependent reduction of SNAP29 in SNc DA neurons from human post-mortem brain tissue of Lewy body pathology (LBP) cases. In summary, our results thus demonstrate a previously unknown effect of α-Syn on intracellular autophagy-associated SNARE proteins and, as a consequence, a reduced autolysosome fusion. As such, our findings will therefore support the investigation of autophagy-associated pathological changes in PD

## Introduction

Macroautophagy (here referred to as autophagy) is a genetically regulated intracellular nutrient recycling process involving lysosomal degradation of unwanted cellular proteins and defective organelles. Autophagy occurs at a low basal level in almost all cell types under the inhibition of the mammalian target of rapamycin complex 1 (mTORC1), a key regulator of autophagy, to sustain cellular homeostasis (rev. in Park et al. [1]). During autophagy, a small cisterna, called the isolation membrane (phagophore), elongates, and surrounds a part of the cytoplasm to form a double-membraned structure, called the autophagosome. Autophagosomes either fuse with late endosomes to form amphisomes, which then fuse with lysosomes, or they fuse directly with lysosomes to form autolysosomes [2]. The formation of autophagosomes and autolysosomes is orchestrated by multimeric protein complexes, including the unc-51-like kinase 1 (ULK1) complex in addition to numerous autophagy-regulated genes (ATGs). Two main signaling molecules, mTORC1 and AMP-activated protein kinase (AMPK), conversely regulate autophagy initiation by phosphorylating ULK1 at different sites [3]. Autophagosomes are thought to form randomly throughout the cytoplasm, before moving to the perinuclear region on microtubule tracks, where the fusion with lysosomes takes place [4]. In cooperation with Ras-related protein in brain (Rab) proteins (Rab7, 33b, 2), ATG8 family proteins, membrane-tethering factors (HOPS, ATG14), and regulatory molecules (RILP, TECPR1, BRUCE, PLEKHM1, Pacer), soluble N-ethylmaleimide sensitive factor attachment protein receptors (SNAREs) eventually mediate the fusion of autophagosomes and amphisomes to lysosomes [5].

Parkinson’s disease (PD) is the most common neurodegenerative movement disorder characterized by the gradual appearance of intraneuronal protein aggregates termed Lewy bodies (LBs). These aggregates are composed of vesicular membrane structures and dysmorphic organelles in conjunction with the protein alpha-Synuclein (α-Syn) [6]. α-Syn likely contributes to the demise of dopaminergic (DA) neurons [7] and the cell-to-cell transmission of α-Syn (‘spreading’) is assumed to promote the progression of pathology throughout the nervous system in PD [8]. Similar to other neurodegenerative conditions [9], α-Syn transmission may be mediated by small extracellular vesicles (EVs) including exosomes [10]. In accord, α-Syn has been identified in exosomes in several in vitro models of transient or stable α-Syn overexpression [11], and in human cerebrospinal fluid (CSF) [12]. Exosomes are derived from multi-vesicular bodies (MVBs) of the late endo-lysosomal pathway, where they are formed by inward budding of the limiting membrane into the MVB lumen and are characterized by specific signature marker proteins, such as Alix/AIP1, Flotillin-1, or CD81 [13]. Various proteins including α-Syn may be targeted to exosomes during their biogenesis in MVBs. In line, α-Syn was shown to associate with the MVB compartment at an ultrastructural level, in a process involving the endosomal sorting complex required for transport (ESCRT) complex [14]. Instead of releasing EVs, MVBs and their cargo may be cleared via autophagic degradation by fusing directly with lysosomes or autophagosomes [15]. The autophagosome-lysosome system thus connects endosomal, lysosomal, and secretory pathways [16], acting as a major hub to sort and rout cargo for recycling, degradation, or secretion of proteins, including α-Syn [17, 18]. Furthermore, autophagy-related molecules are associated with PD pathology [19] and α-Syn impaired autophagic protein degradation in PD and in dementia with Lewy bodies (DLB) [18, 20–27]. Conversely, deficiency in autophagy exacerbates α-Syn pathology thus forming a bidirectional pathogenic loop [17, 28–31]. Taken together, these results functionally connect autophagy and PD pathology and investigating the effect of α-Syn on the autolysosome will therefore support the understanding of cell death and disease progression in PD.

Here, we investigate the effect of α-Syn on SNARE-mediated autolysosome fusion. We found that in cultured DA neurons, α-Syn impaired the fusion of autophagosomes and lysosomes, leading to a reduced abundance of autolysosomes and an increased number of EVs. Mechanistically, the effect of α-Syn on autophagy depended on the autophagy-associated v-SNARE protein SNAP29 because α-Syn specifically reduced the abundance of SNAP29. Conversely, SNAP29 knockdown mimicked the effect of α-Syn on autophagy and EV release, whereas SNAP29 overexpression rescued the α-Syn-induced changes. Our results likely have a disease-relevant implication, because in addition to our results from cultured neurons we found a specific and progressive reduction of SNAP29 in SNc DA neurons from human post-mortem brain tissue of cases that had Lewy body pathology (LBP) at different stages.

## Results

### α-Syn attenuates autophagy turnover

**Fig. 1:**
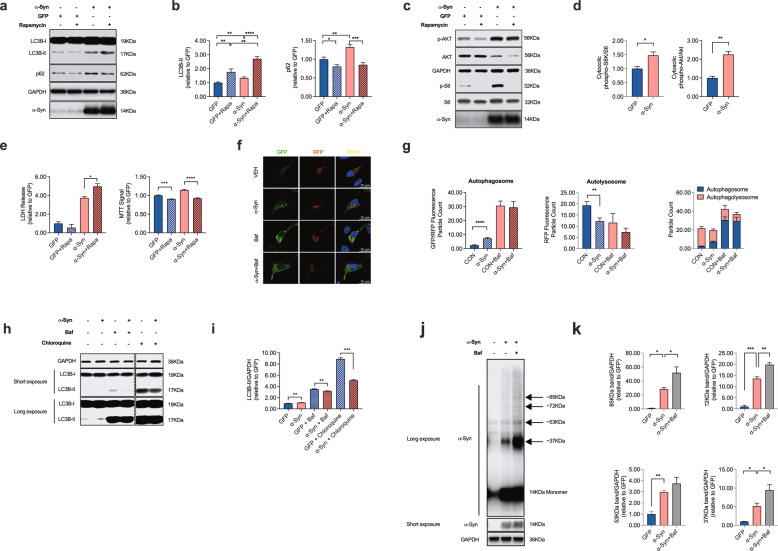
α-Syn overexpression attenuates autophagy turnover. **a**, **b** Western blot and bar graphs illustrating the abundance of sequestome-1/p62 and LC3B-I and -II in response to α-Syn overexpression and in response to treatment with rapamycin (100 nM; 24 h; *n* = 10/condition). **c**, **d** Western blot and bar graphs illustrating the abundance and phosphorylation of mTOR associated signaling molecules (Akt; S6) in response to α-Syn overexpression and in response to treatment with rapamycin (100 nM; 24 h; *n* = 3/condition). **e** Bar graphs illustrating the quantification of LDH in the culture medium (left) and the MTT signal (right) in response to α-Syn overexpression or to treatment with rapamycin (100 nM; 24 h; *n* = 4/condition). **f** Photomicrographs from confocal microscopy of neurons transduced with GFP-RFP-LC3B and either co-transfected with vehicle (VEH), α-Syn or treated with bafilomycin A1 (Baf; 100 nM; 8 h; for VEH *n* = 25 cells, for α-Syn *n* = 26 cells, for Baf *n* = 5 cells, and for Baf+α-Syn *n* = 5 cells) **g** Bar graphs illustrating the count of fluorescence positive particles. α-Syn and Baf both lead to a significant increase in GFP/RFP fluorescence positive particles (left graph), whereas RFP-fluorescence positive particles was decreased (middle graph). The ratio of GFP/RFP double-positive autophagosomes to RFP-positive autolysosomes is decreased in response to α-Syn and Baf (right graph). **h**, **i** Western blot and bar graphs illustrating the increased abundance of LC3B-II in response to Baf (100 nM; 8 h; *n* = 7/condition) or CQ (50 μM, 24 h; *n* = 7/condition). **j** Western blot illustrating the abundance of monomeric and oligomeric α-Syn in response to treatment with Baf (100 nM; 8 h; *n* = 3/condition). **k** Bar graphs depicting the abundance of specific oligomeric α-Syn bands (at 37, 53, 72, 85 kDa) in response to α-Syn, Baf, and both. For comparison of the means, a two-tailed unpaired *t*-test was used in panel **b**, **d**, **g**, **i**; a one-way ANOVA with Šidák’s test for multiple comparisons was used in panel **e**. ****P < 0.0001, ***P < 0.005, **P < 0.01, *P < 0.05. Data are shown as means ± SEM.

In order to investigate effect of the PD-associated protein α-Syn on autophagy turnover in DA neurons, we used Lund Human Mesencephalic cells (LUHMES), a human DA cell line that has been shown to acquire a neuronal phenotype upon differentiation [32]. In order to achieve viral overexpression of α-Syn or GFP as a control, adenoviruses serotype 5 (AV5)-α-Syn or AV5-GFP at a multiplicity of infection (MOI) of 2.15 was added to the cell culture medium 24 h after plating as described previously [33]. We first quantified the abundance of LC3B-I and II in response to α-Syn overexpression in these cells. LC3B is a central protein in the autophagy pathway where it contributes to autophagy substrate selection and autophagosome biogenesis. LC3B is a widely used surrogate protein to quantify the abundance of autophagosomes [34]. When we overexpressed α-Syn in LUHMES cells as described previously [33], we found an increase in LC3B-II, reminiscent of an increased abundance of autophagosomes in response to α-Syn (Fig. 1a, b). Likewise, α-Syn increased the abundance of sequestome-1/p62, a substrate of autophagy (Fig. 1a, b). Co-application of the mTOR inhibitor rapamycin (100 nM, 24 h), which activates autophagy and therefore stimulates the generation of autophagosomes, further increased the abundance of LC3B-II but on the other hand, decreased the abundance of p62 (Fig. 1a, b). These results are consistent with α-Syn attenuating autophagy flux and autophagy initiation. In order to further investigate the effect of α-Syn on autophagy initiation, we tested the effect of α-Syn on the phosphorylation level of the mTOR-activating protein kinase B (Akt) and ribosomal protein S6 kinase (S6; Fig. 1c, d). In accord with our previous findings that demonstrated a stimulatory effect of α-Syn on the mTOR signaling pathway [33], we further confirmed α-Syn to increase the phosphorylation level of these proteins, although at the same time α-Syn also led to a decrease of the total Akt protein abundance. Because activating mTOR exerts an inhibitory effect on autophagy initiation, these initial results therefore suggest α-Syn to increase the abundance of autophagosomes by attenuating autophagy turnover or flux without stimulating autophagy initiation. Notably, rapamycin treatment also potentiated α-Syn-induced cell death in LUHMES cells as demonstrated by an increased LDH release and decreased MTT signal in these neurons (Fig. 1e).

Because α-Syn led to an increased abundance of LC3B-II independent from autophagy initiation, we hypothesized α-Syn to increase LC3B-II by impairing autophagy turnover downstream of the autophagosome. In order to test this hypothesis, we next expressed GFP-RFP-LC3B [35] in LUHMES cells together with α-Syn or Vehicle (Fig. 1f, g). This fluorescence-based assay allows to examine the different stages of autophagy flux by quantifying the abundance of autophagosomes and autolysosomes separately. In accord with our western blot data, we found an increased amount of GFP/RFP-positive autophagosomes in response to α-Syn overexpression, whereas the abundance of RFP-positive autolysosomes was decreased (Fig. 1f, g). Inhibiting the fusion of autophagosomes and lysosomes with bafilomycin A1 (Baf; 100 nM, 8 h), which inhibits autolysosome fusion by blocking the vacuolar H^+^-ATPase-mediated lysosome acidification, likewise led to an accumulation of autophagosomes (Fig. 1f, g) and an increased abundance of LC3B-II (Fig. 1h, i). In order to further confirm these results, we also tested the effect of chloroquine (CQ). CQ is an alternate way to block autophagic flux [36]. Similar to the effect of Baf and α-Syn, we found a dose-dependent increase in the abundance of LC3B-II in CQ-treated neurons (Fig. 1h, i and Supplementary Fig. 1a, b). Taken together, these results further suggest that α-Syn attenuates autophagosome-to-lysosome fusion, and as a consequence, leads to the accumulation of autophagosomes and a decreased abundance of autolysosomes. Notably, the increase in LC3B-II was not a mere consequence of neuronal cell death, since treating cultured neurons with the PD-associated neurotoxins 6-hydroxydopamine (6-OHDA; 20 μM), 1-Methyl-4-phenylpyridinium (MPP ^+^; 10 μM) or annonacin (25 nM) [37] did not result in an increased abundance of LC3B-II (Supplementary Fig. 1c), although inducing a dose-dependent cellular death in LUHMES cells (Supplementary Fig. 1d). When we tested the effect of Baf on distinct α-Syn molecular fractions, we found that Baf resulted in an additional increase in oligomeric α-Syn fractions at 37, 53, 72, and 85 kDa (Fig. 1j, k) suggesting that these α-Syn fractions are subjected to autophagic degradation and that inhibiting autophagy flux leads to the accumulation of α-Syn oligomers, which are commonly regarded as particularly relevant for inducing DA neuronal cell death [38].

### Blockage of autophagy enhances the release of EVs

**Fig. 2:**
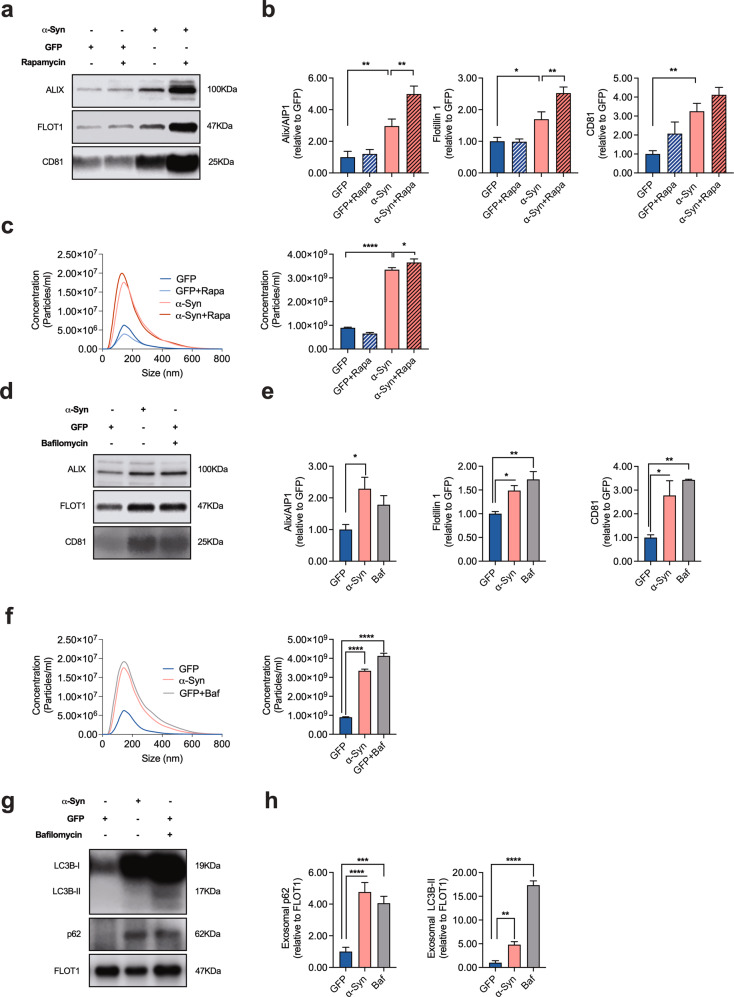
α-Syn overexpression results in an increased release of EVs. **a**, **b** Western blot and bar graphs illustrating the abundance of the EV-associated proteins Alix/AIP1, Flotillin-1, and CD81 in EV-enriched medium pellets from cultured cells in response to α-Syn overexpression and in response to treatment with rapamycin (100 nM; 24 h; *n* = 9/condition). **c** Results from Nanoparticle Tracking Analysis (NTA) illustrating an increased amount of EVs in response to α-Syn overexpression or to treatment with rapamycin (100 nM; 24 h; *n* = 9/condition). **d**, **e** Western blot and bar graphs illustrating the increased abundance of the EV-associated proteins Alix/AIP1, Flotillin-1, and CD81 in EV-enriched medium pellets from cells in response to α-Syn overexpression and in response to treatment with Baf (100 nM; 8 h; *n* = 3/condition). **f** Results from NTA illustrating an increased amount of EVs in response to α-Syn overexpression or to treatment with Baf (100 nM; 8 h; *n* = 9/condition). **g**, **h** Western blot and bar graphs illustrating the increased abundance of LC3B-I, -II, and p62 in EV-enriched medium pellets from α-Syn-transduced or Baf-treated cells. Note that a similar amount of total protein (i.e. a comparable total number of EVs) has been loaded on each lane. The result thus represents the relative content of LC3B and p62 per vesicle. For comparison of the means, a one-way ANOVA with Šidák’s test for multiple comparisons was used in panel **b**, **c**, **e**, **f**, **h** ****P < 0.0001, ***P < 0.005, **P < 0.01, *P < 0.05. Data are shown as means ± SEM.

Since autophagy and the release of EVs are tightly connected [39–41], we next assessed the abundance of the EV-associated proteins Alix/AIP1, Flotillin-1, and CD81 in EV-enriched medium pellets in response to α-Syn overexpression or treatment with rapamycin. We found α-Syn to increase the abundance of EV-associated proteins in the culture medium and that co-application of rapamycin further potentiated this effect for Alix/AIP1 and Flotillin 1, thus mirroring the effect of α-Syn and rapamycin on LC3B-II (Fig. 2a, b). In accord with these results, we found an increased number of extracellular particles in the medium of cells that overexpress α-Syn as measured by Nanoparticle Tracking Analysis (NTA), thus further confirming a stimulatory effect of α-Syn and rapamycin on EV release (Fig. 2c). Conversely, the PD-associated neurotoxic agents 6-OHDA (20 μM, 24 h), MPP ^+ ^(10 μM, 24 h), or annonacin (25 nM; 24 h) failed to induce an increase in EV release (Supplementary Fig. 1e, f), thus suggesting an α-Syn-specific effect that occurs independent from neuronal death.

Similar to α-Syn, the application of Baf resulted in a significant increased abundance of the EV-associated proteins Flotillin-1 and CD81 in EV-enriched medium pellets from α-Syn-transduced neurons as compared to GFP-transduced cells (Fig. 2d, e) and an increased amount of extracellular particles in the culture medium (Fig. 2f). When we investigated the protein composition of EVs, we found that EVs from α-Syn-transduced or Baf-treated neurons carried an increased amount of the autophagy-associated proteins LC3B-I and -II and sequestome-1/p62, thus suggesting their origin from the autophagy pathway (Fig. 2g, h). In addition, α-Syn overexpression led to an increased proportion of oligomeric α-Syn molecular fractions in EV-enriched medium pellets (Supplementary Fig. 1g). Our results are thus consistent with an increased excretion of autophagy-associated intracellular organelles in response to α-Syn-mediated autophagy blockage.

### α-Syn overexpression alters the composition of the autophagolysosomal SNARE complex by affecting SNAP29

**Fig. 3:**
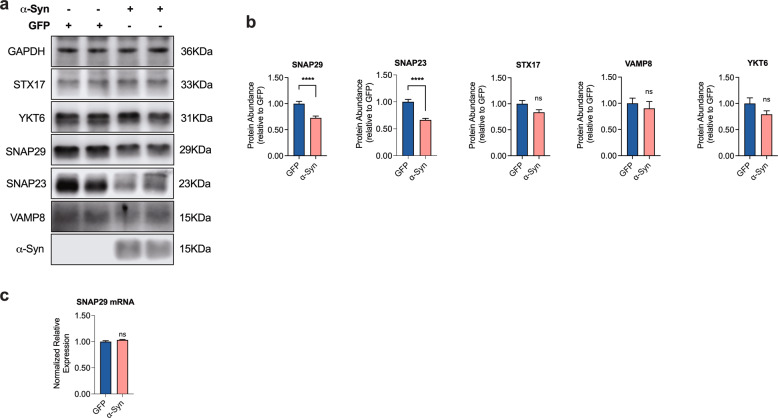
α-Syn overexpression reduces the abundance of SNAP29 in cultured human DA neurons. **a**, **b** Western blot and bar graphs illustrating the SNARE proteins STX17, YKT6, VAMP8, SNAP29, and SNAP23 in α-Syn- and GFP-transduced neurons. α-Syn overexpression specifically leads to a decreased protein abundance of the SNAP25 family members SNAP23 and SNAP29, whereas the other SNARE proteins remain unchanged (*n* = 6/condition, *n* = 11 for SNAP29). **c** Bar graph showing similar levels of SNAP29 mRNA expression in α-Syn-transduced neurons as compared to GFP-transduced cells (*n* = 3/condition). For comparison of the means, a two-tailed unpaired t-test was used in **b**, **c**; ****P < 0.0001; Data are shown as means ± SEM.

In principal, the fusion of autophagosomes and lysosomes depends on a set of specific SNARE molecules, where the autophagic Qa-SNARE syntaxin 17 (STX17) forms a SNARE core complex with the cytosolic Qbc-SNARE SNAP29 and the lysosomal R-SNARE VAMP8 or YKT6 [42–47]. Because α-Syn is well known to interact with synaptic SNARE proteins [48, 49], we therefore hypothesized that α-Syn may impair autophagosome-to-lysosome fusion by affecting one or more of these SNARE molecules. When we examined the abundance of the autophagy-associated SNARE molecules in cultured neurons in response to α-Syn overexpression, we found indeed a significant decrease of the SNAP25 SNARE family members SNAP23 and SNAP29 in α-Syn-transduced cells (Fig. 3a, b). On the other hand, the abundance of VAMP8, STX17, and YKT6 remained unchanged (Fig. 3a, b), thus suggesting a family-specific effect of α-Syn. Interestingly, α-Syn overexpression had no effect on SNAP29 gene expression as measured by qRT-PCR, rather suggesting a posttranscriptional effect of α-Syn on SNAP29 abundance (Fig. 3c).

### The effect of α-Syn on autolysosome fusion depends on SNAP29

**Fig. 4:**
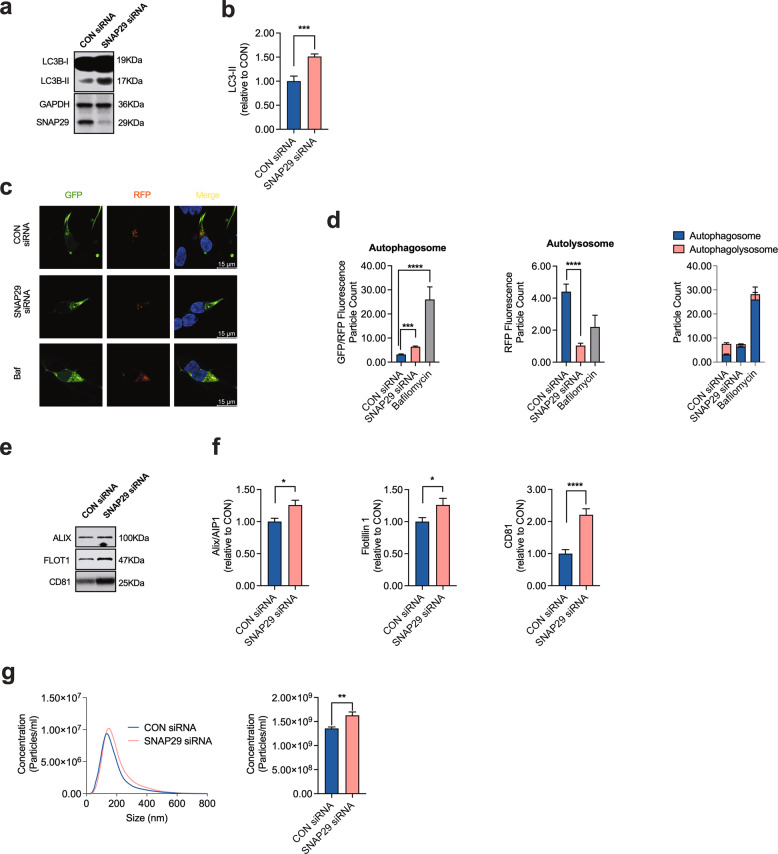
Knocking down SNAP29 mimics the effect of α-Syn on autophagy turnover. **a, b** Western blot and bar graphs illustrating an increased abundance of LC3B-II in response to transfection with SNAP29 siRNAs (30 nM; *n* = 4/condition). **c** Photomicrographs from confocal microscopy of neurons transduced with GFP-RFP-LC3B and either co-transfected with SNAP29 or control (scrambled) siRNAs or treated with Baf (for CON siRNA *n* = 120 cells, for SNAP29 siRNA *n* = 123 cells, for Baf *n* = 5 cells). **d** Bar graphs illustrating the count of fluorescence positive particles. SNAP29 siRNA led to a significant decrease in RFP fluorescence positive particles (middle graph), whereas GFP/RFP-fluorescence positive particles remained unchanged (left graph). The ratio of GFP/RFP double-positive autophagosomes to RFP-positive autolysosomes is decreased in response to SNAP29 siRNA transfection. **e**, **f** Western blot and bar graphs illustrating the increased abundance of the EV-associated proteins Alix/AIP1, Flotillin-1, and CD81 in EV-enriched medium pellets from cells in response to SNAP29 knockdown (*n* = 9/condition). **g** Results from NTA illustrating an increased amount of EVs in response to transfecting cells with SNAP29 siRNAs (*n* = 9/condition). For comparison of the means, a two-tailed unpaired *t*-test was used in panel **b**, **f**, **g**; a one-way ANOVA with Šidák’s test for multiple comparisons was used in panel **d**. ****P < 0.0001, ***P < 0.005, **P < 0.01, *P < 0.05. Data are shown as means ± SEM.

**Fig. 5:**
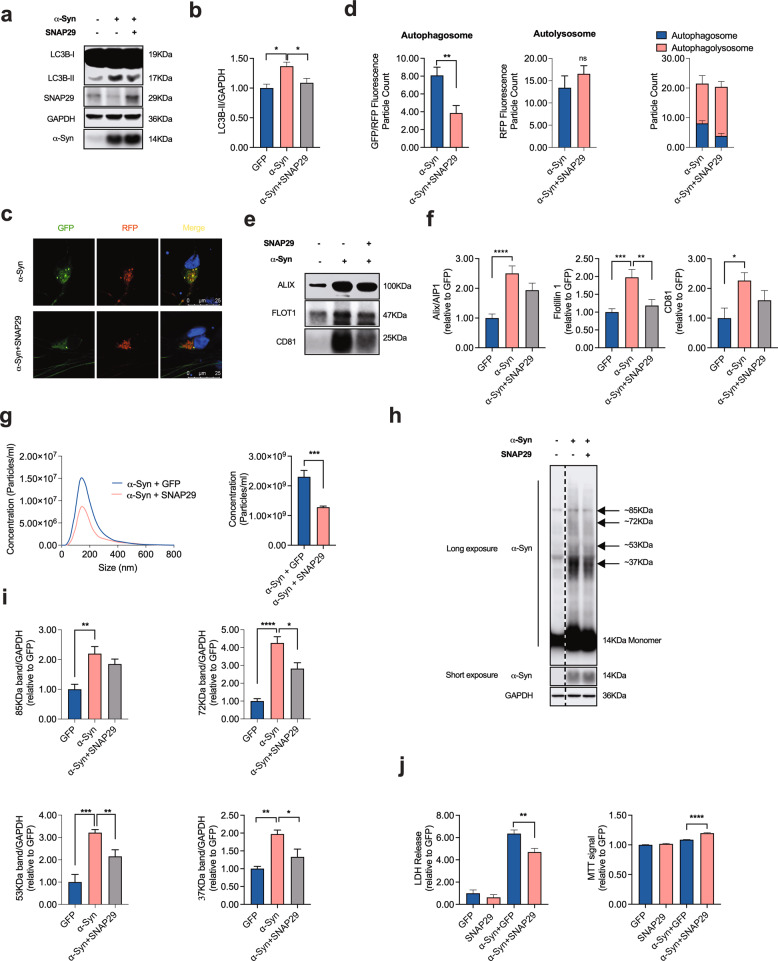
SNAP29 co-expression rescues the α-Syn-induced impairment of autophagy turnover. **a**, **b** Western blot and bar graph illustrating a decreased abundance of LC3B-II in α-Syn overexpressing cells in response to transfection with SNAP29 (*n* = 9/condition). **c** Photomicrographs from confocal microscopy of neurons transduced with GFP-RFP-LC3B, α-Syn and either with SNAP29 or vehicle (VEH). **d** Bar graphs illustrating the count of fluorescence positive particles. SNAP29 co-expression led to a significant decrease in GFP/RFP fluorescence positive particles (left graph), whereas RFP-fluorescence positive particles remained unchanged (middle graph). The ratio of GFP/RFP double-positive autophagosomes to RFP-positive autolysosomes is increased in response to SNAP29 transduction (for α-Syn *n* = 25 cells, for α-Syn + SNAP29 *n* = 28 cells). **e**, **f** Western blot and bar graphs illustrating the decreased abundance of the EV-associated proteins Alix/AIP1, Flotillin-1, and CD81 in EV-enriched medium pellets from cells in response to SNAP29 co-expression (*n* = 9/condition). **g** Results from NTA illustrating a decreased amount of EVs in response to co-transducing cells with SNAP29 (*n* = 9/condition). **h**, **i** Western blot and bar graphs illustrating the abundance of monomeric and oligomeric α-Syn in response to SNAP29 co-expression (*n* = 3/condition). **j** Bar graphs illustrating the quantification of LDH in the culture medium (left) and the MTT signal (right) in response to α-Syn and SNAP29 expression (*n* = 8/condition). For comparison of the means, a two-tailed unpaired *t*-test was used in panel **d**, **g**; a one-way ANOVA with Šidák’s test for multiple comparisons was used in panel **b**, **f**, **i**, **j**. ****P < 0.0001, ***P < 0.005, **P < 0.01, *P < 0.05. Data are shown as means ± SEM.

Based on our previous results, it would be conceivable that α-Syn impairs autophagosome-to-lysosome fusion by affecting SNAP29, and as a consequence the functional integrity of the autolysosomal SNARE complex. In order to test this hypothesis, we next investigated the effect of knocking-down SNAP29 on autophagy turnover. As expected from the role of SNAP29 for autolysosome fusion [43], we found that SNAP29 knockdown significantly increased the abundance of LC3B-II in cultured DA neurons (Fig. 4a, b). Knocking down STX17, another key member of the autolysosomal SNARE complex likewise increased LC3B-II (Supplementary Fig. 2a, b), thus supporting the validity of our model. Similar to α-Syn and Baf treatment (100 nM; 8 h) overexpression, SNAP29 knockdown significantly increased the number of autophagosomes and, at the same time, decreased the abundance of autolysosomes, thus increasing the ratio of autolysosomes to autophagosomes (Fig. 4c, d). SNAP29 knockdown likewise significantly increased the abundance of EV in the culture medium, although the effect on EV releases appears to be smaller as compared to α-Syn, suggesting additional mechanisms by which α-Syn may enhance EV release (Fig. 4e–g). Again, the latter effect was mimicked by knocking down STX17 as a control (Supplementary Fig. 2c, d). Taken together, these results are consistent with SNAP29 knockdown mimicking the effect of α-Syn on autophagy turnover. In order to further establish the functional interplay between SNAP29 and α-Syn, we next tested the effect of SNAP29 co-expression in α-Syn-transduced neurons. Consistent with our previous results, co-expression of SNAP29 normalized the α-Syn-mediated increase in LC3B-II, thus indicating a restored autophagy flux under these conditions (Fig. 5a, b). Likewise, SNAP29 co-expression partially rescued the α-Syn-mediated increase in autophagosomes and normalized the ratio between autophagosomes and autolysosomes without changing the number of autolysosomes (Fig. 5c, d). Interestingly, SNAP29 co-expression likewise tended to normalize the α-Syn-mediated increase of in Alix/AIP1, Flotillin-1, and CD81 in EV-enriched medium pellets (although this effect did not reach statistical significance for Alix/AIP1 and CD81; Fig. 5e, f) and restored the number of EVs in the culture medium (Fig. 5g). Because inhibiting autophagy flux appeared to enhance the generation of oligomeric α-Syn (Fig. 1j, k), we also examined the abundance of α-Syn oligomers in response to SNAP29 and found that co-expressing SNAP29 significantly reduced the abundance of oligomeric α-Syn fractions (Fig. 5h, i). In accord with the hypothesis that oligomeric α-Syn contribute to DA neuronal cell death, we found that co-expressing SNAP29 also attenuated cellular death in α-Syn-transduced neurons (Fig. 5j). In summary, these results suggest that the loss of SNAP29 in α-Syn-transduced neurons contributes to α-Syn-associated neuronal death, possibly through modulating the composition of oligomeric α-Syn.

### α-Syn directly interacts with SNAP29

**Fig. 6:**
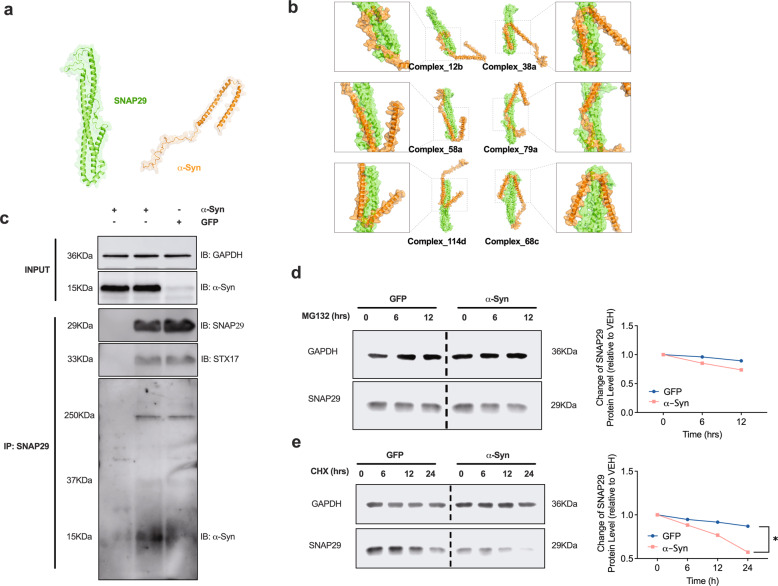
Computational modeling and Co-IP suggest a physical interaction between α-Syn and SNAP29 in cultured neurons. **a** Schematic illustrating the computationally modeled tertiary structure of SNAP29 and α-Syn. **b** Schematic illustrating potential biding sites and complexes between SNAP29 and α-Syn. **c** Western blot illustrating the result of a Co-IP with SNAP29 as a bait. Reacting the membrane with an antibody against α-Syn revealed a clearly visible band in α-Syn-transduced neurons at around 15 kDa. The left lane represents a negative control (no SNAP29 antibody during IP). Representative result from 3 independent experiments. **d** Western blot and graph illustrating the change in SNAP29 protein levels in response to treatment (6 or 12 h) with the proteasome inhibitor MG132 (100 nM). E Western blot and bar graph illustrating the change in SNAP29 during treatment with the protein synthesis inhibitor cycloheximide (CHX; 100 μg/ml). Note the reduced half-life of SNAP29 in the presence of CHX. For comparison of the means, a two-tailed unpaired *t*-test was used in panel **d**, **e**; *P < 0.05. Data are shown as means ± SEM.

Because of our results demonstrating a decrease of SNAP29 in response to α-Syn and since α-Syn has been suggested to directly interact with SNARE complex proteins at presynaptic sites [48], we hypothesized SNAP29 likewise to interact with α-Syn. In order to examine such a protein–protein interaction (PPI) and since the complete tertiary structure of SNAP29 has not been established before, we first generated a computationally modeled structure of SNAP29 (Fig. 6a). SNAP29 domains were first predicted as independent folding units. Thereafter, the units were assembled into full chain models and 5 top-scored structure models were returned and optimized to increase the Rosetta score of these models. Among the five SNAP29 models, #4 showed a good PROCHECK (Ramachandran plot: 95.2% most favored), Verify 3D (93.41% residues were in allowed regions), ERRAT (95.95), and ProSA analysis result (*z*-score: −6.49; Supplementary Fig. 3). These results confirmed the quality of our predicted model of SNAP29. Thus, we select model #4 for the subsequent docking analysis (Fig. 6a). Thereafter we performed a flexible protein-protein docking analysis using protein docking prediction. A total of 492 docking complexes were identified, and the representative docking complexes were evaluated using jsPISA. The analysis showed a low binding energy for all six representative possible docking poses (Fig. 6b and Table 1). These bioinformatical results support a sufficiently strong binding between SNAP29 and α-Syn and in principal support a potential PPI between the two molecules. In order to further validate our bioinformatic analysis with experimental evidence, we conducted a co-immunoprecipitation (Co-IP) with SNAP29 as a bait in neurons that were transduced with α-Syn or GFP (Fig. 6c). In order to better preserve weak or transient PPIs, the cells were treated with formaldehyde, a mild and reversible crosslinker with a very short spacer length (2.3–2.7 Å) that selectively cross-links closely associated proteins. Western blot confirmed a pulldown of α-Syn with SNAP29, thus demonstrating a physical binding of α-Syn and SNAP29 in cultured DA neurons. In summary, these results further support a relevant PPI between α-Syn and SNAP29. Because α-Syn interacting with SNAP29 may promote its proteasomal degradation, we tested the effect of the proteasome inhibitor MG132 (100 nM) on SNAP29. Treating LUHMES cells with MG132 for 6 or 12 h. did not increase the abundance of SNAP29, thus suggesting that SNAP29 is not subjected to proteasomal degradation (Fig. 6d). Conversely, application of the protein synthesis inhibitor cycloheximide (CHX; 100 μg/ml; 6, 12 or 24 h) promoted the decay of SNAP29 in α-Syn-transduced cells (Fig. 6e). In conclusion, these results suggest that SNAP29 has a shorter half live in the presence of α-Syn, but is not subjected to proteasomal degradation.

**Table 1:** Table depicting the bioenergetic and biophysical characteristics of the indicated complexes between SNAP29 and α-Syn.

	Complex_12b	Complex_38a	Complex_58a	Complex_79a	Complex_114d	Complex_68c
Interface area, Å²	986.5	1389	1243	1303	1310	1582
Solvation energy, kcal/mol	−7.633	−15.05	−18.92	−16.09	−16.55	−16.94
Total binding energy, kcal/mol	−12.6	−20.17	−21.88	−22.84	−21.88	−23.39
Hydrophobic *P*-value	0.6641	0.6599	0.4065	0.472	0.5421	0.6159
Number of hydrogen bonds	7	4	5	11	12	12
Number of salt bridges	5	9	2	5	0	3
Number of disulfide bonds	0	0	0	0	0	0

All complexes exhibit a low total binding energy, thus demonstrating an energetically favorable binding between SNAP29 and α-Syn.

### SNAP29 is lost in human SNc neurons from LBP cases

**Fig. 7:**
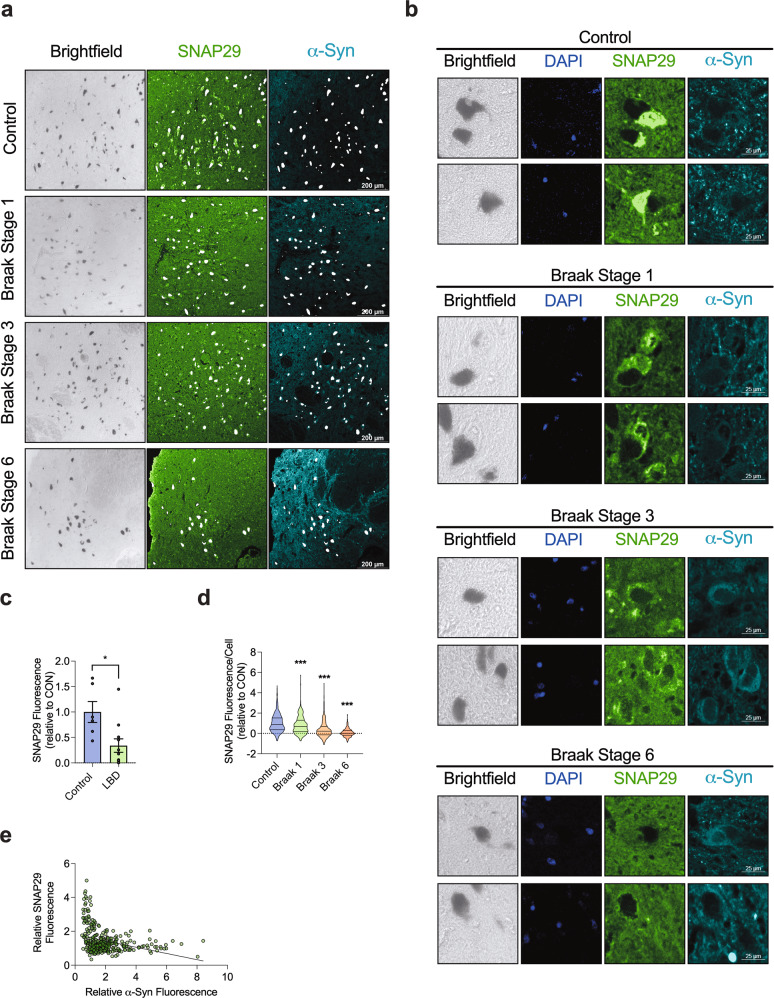
The abundance of SNAP29 is stage-dependently decreased in neuromelanin-positive neurons from LBP cases. **a** Representative photomicrograph from immunohistochemical staining of SNc post-mortem brain tissue at a low magnification (bar: 200 μm). Bright field images indicate neuromelanin pigment (black) in SNc DA neurons. Tissue sections were stained with an antibody against SNAP29 (green) and human α-Syn (blue), revealing a cytoplasmic staining pattern for SNAP29 in neuromelanin-positive neurons. 1st row: pictures from a control case, that had no LBP, 2nd to 4th row: pictures from cases that has LBP at different Braak stages. **b** Representative photomicrograph from immunohistochemical staining of SNc post-mortem brain tissue at a high magnification (bar: 25 μm) from healthy control cases (1st row) and from cases that has LBP at different Braak stages (2nd to 4th row). Tissue sections were stained with an antibody against SNAP29 (green), revealing a cytoplasmic staining pattern for SNAP29 in neuromelanin-positive neurons. Note a stage-dependent decrease of cytoplasmic SNAP29 fluorescence in LBP, whereas control neurons show a clear cytoplasmic SNAP29 fluorescence signal. **c** Bar graph illustrating the average abundance of SNAP29 in LBP cases including all Braak stages (1–6). The average fluorescence signal was analyzed per case (*n* of cases per condition; control: *n* = 6; Braak stage 1: *n* = 3; Braak stage 3: *n* = 2; and Braak stage 6 *n* = 6) **d** Bar graph illustrating a drop of SNAP29 in LBP cases. Different from **c**, the fluorescence signal was analyzed and computed in each neuromelanin-positive cell individually (*n* of cells per condition; control: *n* = 679; Braak stage 1: *n* = 440; Braak stage 3: *n* = 381; and Braak stage 6 *n* = 612), demonstrating a stage-dependent decline of SNAP29 fluorescence in SNc DA neurons of LBP cases. **e** Graph illustrating the correlation of the SNAP29 and α-Syn fluorescence signal in individual SNc neurons (*n* = 352 cells). For comparison of the means, a two-tailed unpaired *t*-test was used in panel **c**; a one-way ANOVA with Šidák’s test for multiple comparisons was used in panel **d**. A linear regression was calculated in panel **e**. ***P < 0.005, *P < 0.05. Data are shown as means ± SEM.

In addition to our experiments in cultured DA neurons, we finally investigated the abundance of SNAP29 in SNc DA neurons in post-mortem human brain tissue from patients with LBP at different stages and in control cases. All cases were examined and staged for LBP by an experienced neuropathologist (T.A.) based on the Braak staging and the recommendations of Brain Net Europe [50] (Table 2). The average tissue fixation time was comparable in control and LBD cases and tissue fixation time had no effect on the SNAP29 fluorescence signal (Supplementary Fig. 4). We next quantified SNAP29 in the cytoplasm of neuromelanin-positive neurons of randomly selected midbrain sections. In control cases that had no LBP, almost all neuromelanin-positive cells had a robust SNAP29 staining (Fig. 7a, b). In contrast, neuromelanin-positive cells from cases that had LBP exhibited a decrease of SNAP29 in SNc neurons (Fig. 7a, b). When we analyzed the average fluorescence value per case, we found a significantly reduced SNAP29 fluorescence in LBP cases vs. control cases (Fig. 7c). Analyzing SNAP29 fluorescence in each DA neurons individually furthermore demonstrated a stage-dependent decrease of SNAP29 in LBP cases (Fig. 7d). Interestingly, we found the decreased abundance of SNAP29 to be already present at Braak stage 1, where Lewy bodies are per definition restricted to the dorsal motor nucleus of the vagus nerve (DMV; Fig. 7a, b, d). When we correlated the abundance of α-Syn with SNAP29 in post-mortem brain tissue, we found a highly significant negative correlation of the two proteins (slope = −0.1838 ± 0.03046; *R*^2^ = 0.1013; *P* < 0.0001), suggesting that α-Syn exerts a dose-dependent effect on SNAP29 in the human SNc (Fig. 7e). These results thus suggest the decrease of SNAP29 as an early pathological event during the progression of Lewy pathology in PD that may precede the appearance of LBs in SNc DA neurons. In accord with our results from cultured cells (Fig. 3), the decay of SNAP29 in human tissue is not an unspecific consequence of neuronal cell death, because staining tissue sections with antibodies against the SNARE complex members STX17 and VAMP8 revealed a readily detectable staining across all stages (Supplementary Fig. 5).

**Table 2:** Neuropathological characterization of brain tissue donors.

Case #	Braak stage	Age at disease onset (years)	Clinical symptoms	Age at death (years)	Sex
*LBD*					
1	5	Unknown	PD, D, Dep	82	f
3	6	Unknown	PD	86	f
4	3	n.a.	–	54	m
5	3	Unknown	PD	58	m
7	6	65	PD	82	m
8	6	49	PD, D	69	m
10	6	54 (PD), 69 (D)	PD, D	74	m
11	6	73	PD	82	f
32	1	n.a.	RLS	90	f
33	1	n.a.	RLS	95	f
34	1	n.a.	–	65	f
*Control*					
14	0	n.a.	–	70	m
15	0	n.a.	–	60	f
16	0	n.a.	–	59	f
18	0	n.a.	–	60	m
19	0	n.a.	–	82	m
20	0	n.a.	–	73	f

All brain tissue was obtained from the Neurobiobank Munich, Department of Neuropathology, Ludwig Maximilian University, Munich. A total number of 20 cases were selected and staged by a trained neuropathologist (T.A.) in regard to the presence of LBs in the medulla oblongata, the tegmentum at level of locus coeruleus, the substantia nigra (SN), and the amygdala and the neocortex (Co). The table depicts the Braak stage, the age and sex of the respective brain tissue.

*PD* Parkinson’s disease, *D* dementia, *Dep* depression, *RLS* restless legs syndrome, – no neurological or psychiatric symptoms.

## Discussion

Our present work indicates that α-Syn impairs autophagy flux by attenuating SNAP29-mediated autolysosome fusion (Figs. 4 and 5). Our present results therefore expand previous reports that collectively demonstrated an inhibitory effect of α-Syn on autophagy through a number of distinct mechanisms [24, 51, 52]. On the other hand, autophagy deficiency has been shown to exacerbate α-Syn pathology [31, 53], thus forming a bidirectional pathogenic loop in PD. Our results support the mechanistical understanding of α-Syn-mediated pathology by demonstrating the relevance of specific SNARE molecules (i.e. SNAP29) for such a vicious circle.

Previous studies indicated that α-Syn is degraded by autophagy [54] whereas autophagy inhibition blocked α-Syn degradation and potentiated its toxicity [17]. Notably, autophagy inhibition has been shown to increase the secretion of smaller oligomers exacerbating cellular damage [30]. Our results suggest that the loss of SNAP29 in α-Syn-transduced neurons contributes to α-Syn-associated neuronal death possibly through modulating the composition of oligomeric α-Syn because oligomeric α-Syn becomes more abundant with autophagy inhibition (Fig. 1j) and is reduced with SNAP29 overexpression (Fig. 5h, i). One could therefore speculate that rescuing SNAP29 protects DA neurons from an increased abundance of ‘toxic oligomers’, as it has been suggested before [55].

A large number of autophagy-associated proteins were identified in LBs [56] in conjunction with aggregated α-Syn, organelles, and lipid membranes, including structures reminiscent of lysosomes and autophagosomes [6]. Likewise, the majority of LBs in PD were found to be immunoreactive for LC3 and LC3-II levels were markedly elevated in the SNc in PD [57]. In addition to these neuropathological findings, our present data reveal novel and previously unknown specific autophagy-associated molecular changes in the PD brain by demonstrating a stage-dependent decline of SNAP29 in post-mortem brain tissue from LBP cases (Fig. 7). In addition to the overall reduction (Fig. 7a–c), we found less SNAP29 in SNc DA neurons as early as Braak stage 1 (Fig. 7d), where LBs are – by definition – restricted to neurons of the medulla oblongata [50]. Our results therefore suggest the decrease of SNAP29 as an early molecular event during the progression of LBP that may precede and enhance the appearance of LBs in SNc neuron. Our current results thus – in addition to our previous studies [33] – add specific insights into the early pathological changes in PD.

**Fig. 8:**
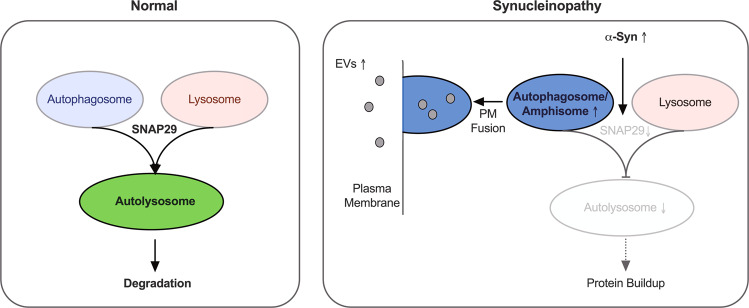
α-Syn impairs autophagosome–lysosome fusion through SNAP29. Graphic summarizing the effect of α-Syn on autophagy turnover. α-Syn overexpression leads to a decreased abundance of SNAP29 which results in an attenuated fusion of autophagosomes with lysosomes. As a result, less autolysosomes are formed and the degradation of cellular materials may be impaired. In order to compensate for a reduced autophagic flux, autophagosomes fuse with the plasma membrane (PM) to release EVs into the extracellular space.

Our results demonstrate an increased release of EVs in α-Syn-transduced neurons (Fig. 2). We found α-Syn-mediated EV release to be functionally connected to autophagy turnover because autophagy induction by rapamycin further increased EV release (Fig. 2a–c) similar to blocking autophagy turnover by Baf or SNAP29 knockdown (Figs. 2d–f and 4e–g). Conversely, enhancing autophagy flux by SNAP29 overexpression led to a reduced abundance of EVs (Fig. 5e–g). Our data thus indicate a model where α-Syn leads to a decrease in SNAP29, a reduced fusion of autophagosomes with lysosomes and, as a consequence, to an increased EV release that possibly compensates for the attenuated degradation of cellular materials by excreting it (Fig. 8). In accord α-Syn-induced EVs carried an increased amount of autophagy-related molecules (Fig. 2g, h), thus suggesting their generation from amphisomes (i.e. MVB/autophagosome hybrid organelles) [41]. When defects in autophagy or lysosomal function prevent the efficient degradation of intracellular protein aggregates, exosomal α-Syn release may be enhanced to alleviate proteotoxic stress [41]. Ironically, this behavior might be responsible for the propagation of the disease phenotype when neighboring neurons take up these exosomes.

SNARE-complexes drive the fusion of membrane-bound vesicles with target membranes or with each other. The SNAP-25 protein sub-family includes both the Qb- and Qc SNARE-domains within a single protein. Autophagosome-lysosome fusion is known to be mediated by specific SNARE molecules, including STX17, VAMP7/8, SNAP29, STX17, and YKT6 [43, 46, 47]. Notably, previous reports identified two distinct SNARE complexes to mediate autophagosome/lysosome fusion: STX17–SNAP29–VAMP7/8 [43, 58] and YKT6–SNAP29–STX7 [47], thus identifying SNAP29 as an ubiquitary SNARE protein implicated in different pathways of autophagosome/lysosome fusion. In vertebrates, the SNAP-25 sub-family consists of SNAP-25, SNAP-23, SNAP-29, and SNAP-47, named for their apparent molecular weights. Whereas SNAP-25 and SNAP-23 are specialized for driving regulated exocytosis, SNAP-29 is present on intracellular membranes and performs functions in autophagosome-to-lysosome fusion [59]. Our results from investigating the abundance of SNAP23 and SNAP29 demonstrated a reduction for both in α-Syn-transduced neurons, thus suggesting a family-specific effect of α-Syn (Fig. 3a, b). Since α-Syn has been shown to physically interact with synaptic SNARE proteins chaperoning their assembly [48], a possible scenario would be that α-Syn physically interacts with SNAP29 to facilitate its degradation. Indeed, our PPI modeling and Co-IP experiments all suggest α-Syn to physically interact and bind to SNAP29 in cultured neurons (Fig. 6). In addition, our results argue against a proteasomal degradation of SNAP29, but do not reveal the specific mechanism by which such a presumed SNAP29 complex may be degraded. Future studies should therefore address the specific intermolecular interaction between both molecules and the consequences of such an interaction for the abundance of SNAP29, where the particular molecular domains of SNAP25 family members with dual Qb and Qc SNARE motifs may provide a structural basis for specific binding. Finally, such prospective studies will have to segregate the specific cellular effects of α-Syn-induced SNAP29 loss on autophagy from additional SNAP29-dependent mechanisms, such as its recently described role in the ER and Golgi apparatus, where it promotes secretory vesicle trafficking [1, 60].

## Methods

### LUHMES cell culture

Proliferating Lund human mesencephalic (LUHMES) cells were maintained in Thermo Scientific Nunc EasYFlask™ Cell Culture Flasks, while differentiated LUHMES cells were plated in Thermo Scientific Nunc™ Cell-Culture Dishes/Multidishes (Thermo Fisher Scientific). Proliferating and differentiated LUHMES cells were cultured at 37 °C with 5% CO_2_ and water-saturated air. The proliferation medium consisted of Dulbecco’s modified Eagle’s medium/nutrient mixture F-12 Ham (DMEM/F-12, Sigma-Aldrich) with 1% N-2 supplement (Life Technologies) and 0.04 µg/ml recombinant human FGF-basic (Peprotech). The differentiation medium consisted of DMEM/F-12 supplemented with 1% N-2 supplement, 1 µg/ml tetracycline (Sigma-Aldrich), 0.5 µg/ml N6,2′-O-Dibutyryladenosine 3′,5′-cyclic monophosphate sodium salt (Dibutyryl cyclic-AMP, Sigma-Aldrich), and 2 ng/mL recombinant human GDNF (Bio-Techne). Before plating the cells for proliferating and differentiation, tissue culture vessels were coated with 0.1 mg/mL poly-l-ornithine solution (PLO, Sigma-Aldrich) at 37 °C for 24 h, followed by three times washing with Dulbecco’s phosphate-buffered saline (DPBS, Sigma-Aldrich). As for cell differentiation, cell culture vessels were further coated with 5 µg/ml bovine fibronectin (Sigma-Aldrich) at 37 °C for 24 h. For differentiation, unless stated otherwise, the cells were seeded at a density of 100,000 cells/cm^2^ to achieve a final confluence of ~50%, and thereafter differentiated for 6 days in differentiation medium into post-mitotic neurons with a DA phenotype [33].

In order to achieve viral overexpression of α-synuclein (α-Syn) or GFP as a control, adenoviruses serotype 5 (AV5)-α-Syn or AV5-GFP at a multiplicity of infection (MOI) of 2.15 was added to the cell culture medium 24 h after plating as described previously [33]. As for SNAP29 overexpressing experiments, adenovirus-associated viruses serotype DJ/8 (AAVDJ/8)-SNAP29 or AAVDJ/8-GFP at a MOI of 4000 was added 6 h after AV5-α-Syn application. After the virus application, the cells were incubated for 24 h. Thereafter, virus medium was removed and cells were washed three times with DPBS before fresh differentiation medium was added. Cell culture medium was changed 24 h before harvesting on day 6 of differentiation. For rapamycin or bafilomycin A1 (both Sigma-Aldrich) treatment, the compound was dissolved in DMSO and added to cells at a final concentration of 100 nM after the medium change. The same volume of DMSO was added to cells as a control. The final concentration of DMSO was <0.1% (v/v).

### Small interfering RNA transfection

SNAP29 knockdown was achieved by using small interfering RNAs (siRNA). Therefore, a magnetic nanoparticle transfection kit (NeuroMag Starting Kit, OZ Biosciences) and Silencer Select siRNAs™ targeting SNAP29 (siRNA ID: s17859, Thermo Fisher Scientific) were used according to the manufacturer’s instructions. In brief, SNAP29 siRNAs or Silencer™ Select Negative Control No. 1 siRNAs (# 4390843) were incubated with NeuroMag regent in Optimum medium (Thermo Fisher Scientific) for 15 min at room temperature before the mixture was added to the cells. Thereafter, cell culture vessels were transferred back to the incubator, and placed on a magnetic plate for 30 min to allow transfection.

### LDH assay and MTT assay

Cell death in cultured DA neurons was quantified using lactate dehydrogenase (LDH) assay and 5-diphenyltetrazolium bromide (MTT) assay on day 6 of differentiation. LUHMES cells were seeded in 300 µl differentiation medium per well in 48-well plates, followed by virus transduction and/or rapamycin treatment as described above. For LDH assay, 30 µl of medium of each well was transferred to a 96-well plate and 70 µl of 80 mM Tris/HCl/200 mM NaCl (pH = 7.2) buffer containing 10 mM NADH and 100 mM pyruvate (Sigma-Aldrich) was added. The absorption of NADH at 340 nm was monitored with a reference measurement at 420 nm using a microplate reader (ClarioStar, BMG labtech, Ortenburg, Germany). For the positive control, cells were lysed using Triton™ X-100 (Sigma-Aldrich) for maximal LDH release. The percentage of LDH release was calculated by taking the ratio of LDH released into the supernatant to the total LDH in the supernatant and the cell lysate. For MTT assay, 30 µl MTT (5 mg/ml in sterile DPBS) was added to each well and incubated back in the incubator for 1 h. After careful removal of the medium, the plate was frozen at −80 °C for 1 h. Thereafter, 300 µl of DMSO was added to each well and the plate was put on a plate shaker to homogenize. After the violet crystals were dissolved, the absorption at 590 nm was monitored with a reference measurement at 630 nm using a microplate reader (ClarioStar, BMG labtech, Ortenburg, Germany).

### LC3B-GFP-RFP autophagy reporter assay

The RFP-GFP-LC3B fusion protein was expressed in LUHMES cells using BacMam 2.0 RFP-GFP-LC3B reagent from Premo™ Autophagy Tandem Sensor RFP-GFP-LC3B kit (Thermo Fisher Scientific). This tandem RFP-GFP sensor capitalizes on the pH difference between the acidic autolysosome and the neutral autophagosome, and the exhibited green/red (yellow) or red fluorescence enables the visualization of the autophagosome to autolysosome progression. In brief, LUHMES cells were plated and grown on PLO and fibronectin-coated µ-Slide 8-Well Ibidi chambers (Ibidi) and were transduced and treated as described above. Baculoviral infection was performed on day 4 of differentiation according to the manufacturer’s instructions. The cells were fixed using 4% formaldehyde for 20 min at room temperature on day 6 of differentiation. Pictures were captured under a Leica SP5 confocal microscope (Leica, Wetzlar, Germany). Images were processed and GFP or RFP dots counts were carried out using the open-source image analysis platform FIJI (http://fiji.sc/Fiji). More than 25 cells were analyzed for each condition. The average number of GFP or RFP dots per cell was determined.

### RNA extraction and reverse transcription

For total RNA extraction, a RNeasy Plus Kit was used according to the manufacturer’s protocol (Qiagen). In brief, the cell culture medium was removed and cells were washed with DPBS before 350 µl of Buffer RLT Plus were added to the cells. The cell lysate was transferred into a microcentrifuge tube and homogenized by vortexing for 30 s, followed by centrifugation for 30 s at 8000 × *g* in a gDNA Eliminator spin column. The flow-through was mixed with 350 µl of 70% ethanol, transferred to a RNeasy spin column, and centrifuged for 15 s at 8000 × *g*. Thereafter, the column was washed by adding 700 µl of Buffer RW1 to the column, followed by another centrifugation for 15 s at 8000 × *g*. Subsequently, the column was washed twice with 500 µl of Buffer RPE by centrifugation at 8000 × *g*, for 15 s and 2 min, respectively. The RNeasy spin column was placed in a new 2 ml collection tube and the membrane was dried by centrifugation at full speed for 1 min. After supplying a new 1.5 ml collection tube, RNA was eluted by adding 30 µl of RNase-free water directly to the spin column membrane and centrifugation for 1 min at 8000 × *g*. Total RNA concentration was quantified using a Nanodrop 2000 spectrophotometer (NanoDrop). Reverse transcription was performed using an iScript™ cDNA Synthesis Kit (Bio-Rad Laboratories). For each reverse transcription extraction, 1 µg extracted RNA, 10 µl iScript Reaction Mix, and 2 µl iScript Reverse Transcriptase were used. The reaction was performed using the following protocol: 5 min at 25 °C, 20 min at 46 °C, and 1 min at 95 °C.

### Quantitative real-time PCR

Gene expression of SNAP29 was validated using semi-quantitative real-time PCR (qRT-PCR) in a Step One Plus instrument (Thermo Fisher Scientific). For qRT-PCR analysis, SYBR™ Select Master Mix (Thermo Fisher Scientific), 2.5 ng complementary DNA from total RNA, and 0.2 µM forward and reverse primers were used. The PCR primer sequences are given in the Supplementary Table 1. The reaction was performed according to the following protocol: 2 min at 50 °C, 2 min at 95 °C, and 40 cycles of 15 s at 95 °C and 60 s at 60 °C. The melting curves were recorded, and the cycle threshold (CT) values were set within the exponential phase of the PCR. Four housekeeping genes (ACTB, GAPDH, GPBP1, and RPL22) were tested in total, and two of them (GPBP1 and RPL22) were used for data normalization according to geNorm analysis. Comparative normalized relative quantities (CNRQ) were used to calculate the relative expression levels using qBase Plus software (Biogazelle). Gene expression was statistically evaluated by two-tailed Student’s *t* test on the assumption of equal variances.

### Protein extraction and BCA assay

For total protein extraction from LUHMES cells or extracellular vesicle (EV) enriched medium pellets, samples were lysed in pre-chilled RIPA buffer freshly supplemented with protease and phosphatase inhibitors (Complete™ Protease Inhibitor Cocktail, PhosStop™ Phosphatase Inhibitor Cocktail, both Roche). The lysates were incubated for 30 min on ice and centrifuged at 13,000 × *g* for 15 min at 4 °C. The supernatants were obtained and subjected to the following experiments. BCA Assay was performed to evaluate total protein concentration by using a Pierce™ BCA Protein Assay Kit (Thermo Fisher Scientific) according to the manufacturer’s instructions. Briefly, a BCA working solution was prepared by mixing 50 volume of Reagent A and 1 volume of Reagent B together. Thereafter, the BCA working solution was thoroughly mixed with a sample and incubated for 30 min at 60 °C. The optical density was measured at 562 nm using a Nanodrop 2000 spectrophotometer (NanoDrop).

### Western blot analysis

Total protein concentration was normalized according to BCA assay prior to western blot unless stated otherwise. Thereafter, samples were denatured by heating to 95 °C for 5 min in Laemmli sample buffer containing 10% β-mercaptoethanol, and run on AnykD™ Criterion™ TGX™ precast gels (Bio-Rad Laboratories) with tris-glycine-based running buffer. Proteins were transferred from polyacrylamide gels onto polyvinylidene difluoride (PVDF) membranes using a semi-dry transfer system (Trans-Blot^®^ Turbo™ System, Bio-Rad). Non-specific binding sites were blocked with 5% (w/v) skimmed milk in Tris-buffered saline with 0.05% (v/v) Tween20 (TBST) for 1 h and the membrane was incubated at 4 °C overnight under gentle shaking with the primary antibody in TBST with 5% (w/v) BSA (Cell Signaling Technology). The membrane was washed and incubated with the respective HRP-conjugated secondary antibody (Vector Labs) in TBST for 2 h at room temperature. The protein bands were detected by using Clarity™ Western ECL Substrate Kit (Bio-Rad Laboratories) or ECL Prime™ (GE Healthcare), and LI-COR Odyssey^®^ Fc Imaging system (LI-COR Biosciences). Band intensities were quantified using Image Studio™ software (LI-COR Biosciences). For proteins of interest, band intensities were normalized to the housekeeping protein GAPDH. All antibodies used for western blot are listed in Supplementary Table 2.

### Co-immunoprecipitation

Co-immunoprecipitation assays were performed using a Pierce™ Co-Immunoprecipitation Kit (Thermo Scientific) following the manufacturer’s instructions with subtle modifications. Formaldehyde in-cell crosslinking was performed prior to co-immunoprecipitation. Therefore, the cells were washed once with DPBS before incubated with 1% (w/v) formaldehyde in DPBS for 20 min at room temperature. Thereafter, 1/10 volume of 1.25 M glycine was added to quench crosslinking for 5 min. The cells were washed twice with Modified Dulbecco’s PBS (0.008 M sodium phosphate, 0.002 M potassium phosphate, 0.14 M sodium chloride, and 0.01 M KCl; pH 7) and subsequently lysed in pre-chilled IP Lysis/Wash Buffer (0.025 M Tris, 0.15 M NaCl, 0.001 M EDTA, 1% NP-40, 5% glycerol; pH 7.4) freshly supplemented with protease and phosphatase inhibitors (Complete™ Protease Inhibitor Cocktail, PhosStop™ Phosphatase Inhibitor Cocktail, both Roche). The lysates were incubated for 30 min on ice and centrifuged at 13000 × *g* for 15 min at 4 °C, and supernatants were obtained and the total protein concentrations were normalized according to BCA assay. For Co-IP, 50 µl AminoLink Plus Coupling Resin slurry was transferred to Pierce Spin Columns and incubated with 10 µl of anti-SNAP29 antibody (Abcam) for 2.5 h on a rotator at room temperature for antibody immobilization. For the negative control, Pierce Control Agarose Resin was used in the same conditions. After antibody immobilization, 500 µg of the lysate’s proteins were diluted with IP Lysis/Wash Buffer to a final volume of 500 µL per column and incubated with the resins on a rotator for 6 h at 4 °C. The resin was washed with Modified Dulbecco’s PBS according to the manufacturer’s instructions. Thereafter, 40 µl of the elution buffer was passed through each resin to elute SNAP29 and the associated proteins from the beads. For protein denaturalization, the 5x sample buffer containing 10% β-mercaptoethanol was added to eluted fractions which were subsequently heated at 95 °C for 5 min. Finally, Co-IP fractions as well as the input fractions were subjected to Western blot analysis for SNAP29 and a-Syn detection.

### Fluorescent immunohistochemistry

Formalin-fixed and paraffin embedded human post-mortem brain tissue samples were obtained from the Neurobiobank Munich (Center for Neuropathology, Ludwig Maximilian University, Munich, Germany) with approval by the local ethics committee and in accordance with the anatomical tissue procurement guidelines. Human SNc sections were deparaffinized in xylene and rehydrated in graded ethanol series. Antigen retrieval was performed in sodium citrate buffer for 30 min at 95 °C. Sections were subsequently permeabilized with 0.5% Triton X-100 in PBS for 10 min and blocked in 5% normal serum (Vector Laboratories) for 1 h at room temperature, followed by incubating them with the respective primary antibody overnight at 4 °C in a humidified chamber. Thereafter, tissue sections were washed with PBS and incubated with anti-rabbit biotinylated secondary antibodies (Vector Laboratories) for 2 h at room temperature. After washing with PBS, the sections were incubated with AB solution (ABC kit, Vector) for 1 h, and subsequently with 10 µM biotinylated tyramide containing 0.005% hydrogen peroxide for 20 min for signal amplification [61]. The sections were subsequently incubated with Alexa Fluor™ 488-conjugated streptavidin (Invitrogen) for 2 h. For SNAP29-αSyn double staining, the sections were additionally incubated with αSyn primary antibody overnight at 4 °C, and subsequently incubated with Alexa Fluor® 647 Anti-Rabbit IgG for 2 h. Finally, nuclei were counter-stained with DAPI for 10 min followed by washing and mounting with Fluoroshield™ mounting medium (Sigma-Aldrich). All images were captured using a Leica SP5 confocal microscope with the same setting and analyzed using the open-source image analysis platform FIJI (http://fiji.sc/Fiji). Regions of interest (ROI) of neuromelanin-positive cells were selected manually based on both the brightfield channel and green channel, and the mean SNAP29 fluorescent intensity was quantified. The background fluorescent intensity of SNAP29 in each field was acquired and subtracted from corresponding cell fluorescent intensity for normalization. At least three fields or 30 cells were used for analysis for each case (blinded). The average SNAP29 intensity per case was determined. All antibodies used are listed in Supplementary Table 2.

### Extracellular vesicle isolation

To prepare EV-enriched pellets from cell culture medium, the medium was changed at day 5 of differentiation and was harvested after 24 h. EVs were isolated by differential ultracentrifugation. The cell culture medium was first centrifuged at 300 × *g* for 10 min at 4 °C to pellet cells. The supernatant was then centrifuged at 2000 × *g* for 10 mins and at 10000 × *g* for 30 mins. The supernatant was transferred to ultracentrifuge tubes and centrifuged twice in a TLA-55 rotor (Beckman) at 100,000 × *g* for 90 min at 4 °C. The supernatant was removed and the pellet was resuspended with DPBS between the two ultra-centrifugations. After centrifugation, the supernatant was discarded, and the pellet was lysed in an equal volume of pre-chilled RIPA buffer supplemented with protease inhibitors for further western blot analysis. For autophagy markers blots, the total protein concentration of the lysates was normalized according to BCA assay, while for EV quantification, the lysates were directly subjected to EV markers blots.

### Nanoparticle tracking analysis

For EV size distribution profiles and EV quantification, NTA analysis was carried out as described [62] using a NanoSight LM10 system (Malvern), which analyses particle size based on Brownian motion. Cell culture medium was changed at day 5 of differentiation and was harvested after 24 h. For each sample, three 60-s videos were recorded. Replicate histograms were generated from the videos, using the NanoSight software 3.0 (Malvern), representing mean and confidence intervals of the three recordings for each sample.

### Three-dimensional protein structure prediction and validation

The three-dimensional structure model of α-Syn was obtained from the Protein Data Bank (PDB) database (10.2210/pdb1XQ8/pdb [63], accessed on 03/08/2020). The complete protein structure of the SNAP29 protein was not available in the PDB, thus the structure model of SNAP29 was computationally modeled using Rosetta webserver [64] (http://robetta.bakerlab.org), which predicted protein structure using the Rosetta ab initio and homology comparative modeling structure prediction methods [65, 66]. The sequence of SNAP29 used for the computation was retrieved from UniProt online database (https://www.uniprot.org/uniprot/O95721, accessed on 03/08/2020). The predicted SNAP29 models were further optimized using PyRosetta FastRelax [67–71], and the returned full-atom relaxed structures were evaluated for protein geometry using PROCHECK (Ramachandran plot) [72], VERIFY 3D [73], and ERRAT [74] on the Structure Analysis and Verification Server (SAVES, https://servicesn.mbi.ucla.edu/SAVES), and ProSA-web [75, 76] (https://prosa.services.came.sbg.ac.at/prosa.php). The best SNAP29 model was selected based on the results of the abovementioned evaluations.

### Protein docking simulation

Protein docking simulations were performed using the protein docking prediction server SwarmDock [77–79] (https://bmm.crick.ac.uk/~svc-bmm-swarmdock/index.html), which performed flexible modeling of SNAP29-a-Syn complexes using the SwarmDock algorithm which incorporates a normal modes approach. Properties and inference on probable SNAP29-α-Syn complexes assemblies were evaluated using jsPISA [80, 81] (http://www.ccp4.ac.uk/pisa). The structural figures were produced with an open-source version of Pymol (https://github.com/schrodinger/pymol-open-source).

### Statistical analysis

Prism 7 (GraphPad Software) was used for statistical analysis and for creating line and bar graphs. Two datasets were compared by *t*-tests. When there were more than two datasets, assays with one variable with were compared by one-way ANOVAs with Tukey’s or LSD post hoc test. All data are shown as mean ± SEM. *P < 0.05 was considered to be significant.

## Supplementary information

Supplemental Material

AJ Checklist

Author Contribution Form

## Data Availability

This study includes no data deposited in external repositories.
