# Supplementary material for: Alpha-Synuclein defects autophagy by impairing SNAP29-mediated autophagosome-lysosome fusion
Source: Cell Death Dis. 2021 Sep 17;12(10):854. doi: 10.1038/s41419-021-04138-0 (PMC8448865; doi:10.1038/s41419-021-04138-0)
Supplement: Supplementary file 1 — Supplemental Material [file 41419_2021_4138_MOESM1_ESM.docx]

**Supplementary Material:**

**Alpha-Synuclein defects autophagy by impairing SNAP29-mediated autophagosome-lysosome fusion**

**Qilin Tang, Pan Gao, Thomas Arzberger, Matthias Höllerhage, Jochen Herms, Günter Höglinger & Thomas Koeglsperger**

**Supplementary Table 1:**

| **Gene** | | **Primer** | **Sequence 5' to 3'** |
| --- | --- | --- | --- |
| SNAP29 | Synaptosomal-associated protein, 29kDa | Forward Primer | TCATGTACGAGTCCGAGAAGG |
|  |  | Reverse Primer | CCCAAACACGCTCTTAATGCTAT |
| RPL22 | Ribosomal protein L22 | Forward Primer | CACGAAGGAGGAGTGACTGG |
|  |  | Reverse Primer | TGTGGCACACCACTGACATT |
| GPBP1 | GC-rich promotor  binding protein 1 | Forward Primer | ATCATTCGGTCTTCAACCTTCC |
|  |  | Reverse Primer | ATCCTCAGTTAAGGGAGCACA |
| GAPDH | Glyceraldehyde-3-phosphate dehydrogenase | Forward Primer | TCGGAGTCAACGGATTTGGT |
|  |  | Reverse Primer | CCTGGAAGATGGTGATGGGA |
| ACTB | Actin beta | Forward Primer | TCACCAACTGGGACGACATG |
|  |  | Reverse Primer | GAGGCGTACAGGGATAGCAC |
|  |  |  |  |

**Supplementary Table 1:** Table summarizing all oligonucleotide primers used in the present study.

**Supplementary Table 2:**

| **Antibody** | **Cat. No.** | **Application** | **Dilution** | **Supplier** |
| --- | --- | --- | --- | --- |
| AKT | 9272S | WB | 1:1000 | Cell Signaling Technology |
| Alix (E6P9B) | 92880S | WB | 1:500 | Cell Signaling Technology |
| Alpha-synuclein (14H2L1) | 701085 | WB for co-IP | 1:500 | Thermo Fisher Scientific |
| Alpha-synuclein (C-20) | sc-7011-R | WB | 1:1000 | Santa Cruz |
| Alpha-synuclein (MJFR1) | ab138501 | IHC | 1:100 | Abcam |
| AMPKα (D63G4) | 5832S | WB | 1:1000 | Cell Signaling Technology |
| CD81 (B-11) | sc-166029 | WB | 1:500 | Santa Cruz |
| Flotillin-1 (D2V7J) | 18634S | WB | 1:500 | Cell Signaling Technology |
| GAPDH (6C5) | sc-32233 | WB | 1:1000 | Santa Cruz |
| LC3B (D11) | 3868S | WB | 1:1000 | Cell Signaling Technology |
| Phospho-Akt (Ser473) | 9271S | WB | 1:1000 | Cell Signaling Technology |
| Phospho-AMPKα (Thr172) | 2535S | WB | 1:1000 | Cell Signaling Technology |
| Phospho-S6 Ribosomal Protein Ser240/244 | 2215S | WB | 1:1000 | Cell Signaling Technology |
| S6 Ribosomal Protein (5G10) | 2217S | WB | 1:1000 | Cell Signaling Technology |
| SNAP29 | MAB7869 | WB | 1:250 | R&D Systems |
| SNAP29 [EPR9198(2)] | ab181151 | IHC | 1:100 | R&D Systems |
| SNAP29 [EPR9198(2)] | ab181151 | co-IP | 1:50 | Abcam |
| SQSTM1/p62 (D5L7G) | 88588S | WB | 1:500 | Cell Signaling Technology |
| Syntaxin 17 (D3D7H) | 31261S | WB | 1:1000 | Cell Signaling Technology |
| Syntaxin 17 (D3D7H) | 31261S | IHC | 1:100 | Cell Signaling Technology |
| VAMP8 | 13060S | WB | 1:1000 | Cell Signaling Technology |
| VAMP8 | 13060S | IHC | 1:100 | Cell Signaling Technology |
| YKT6 | PA5-56565 | WB | 1:1000 | Thermo Fisher Scientific |
| Goat Anti-Rabbit IgG Antibody (H+L), Biotinylated | BA-1000 | IHC | 1:200 | Vector |
| Alexa Fluor® 488 AffiniPure Donkey Anti-Rabbit IgG (H+L) | 711-545-152 | IHC | 1:500 | Jackson ImmunoResearch |
| Alexa Fluor® 647 AffiniPure F(ab')₂ Fragment Donkey Anti-Rabbit IgG (H+L) | 711-606-152 | IHC | 1:500 | Jackson ImmunoResearch |
| Peroxidase Labeled Goat anti-Mouse IgG H+L | PI-2000 | WB | 1:5000 | Vector |
| Peroxidase Labeled Goat anti-Rabbit IgG H&L | PI-1000 | WB | 1:5000 | Vector |

**Supplementary Table 2:** Table summarizing all antibodies used in the present study including dilution factor and purchasing information.

**Supplementary Figure 1:**

**
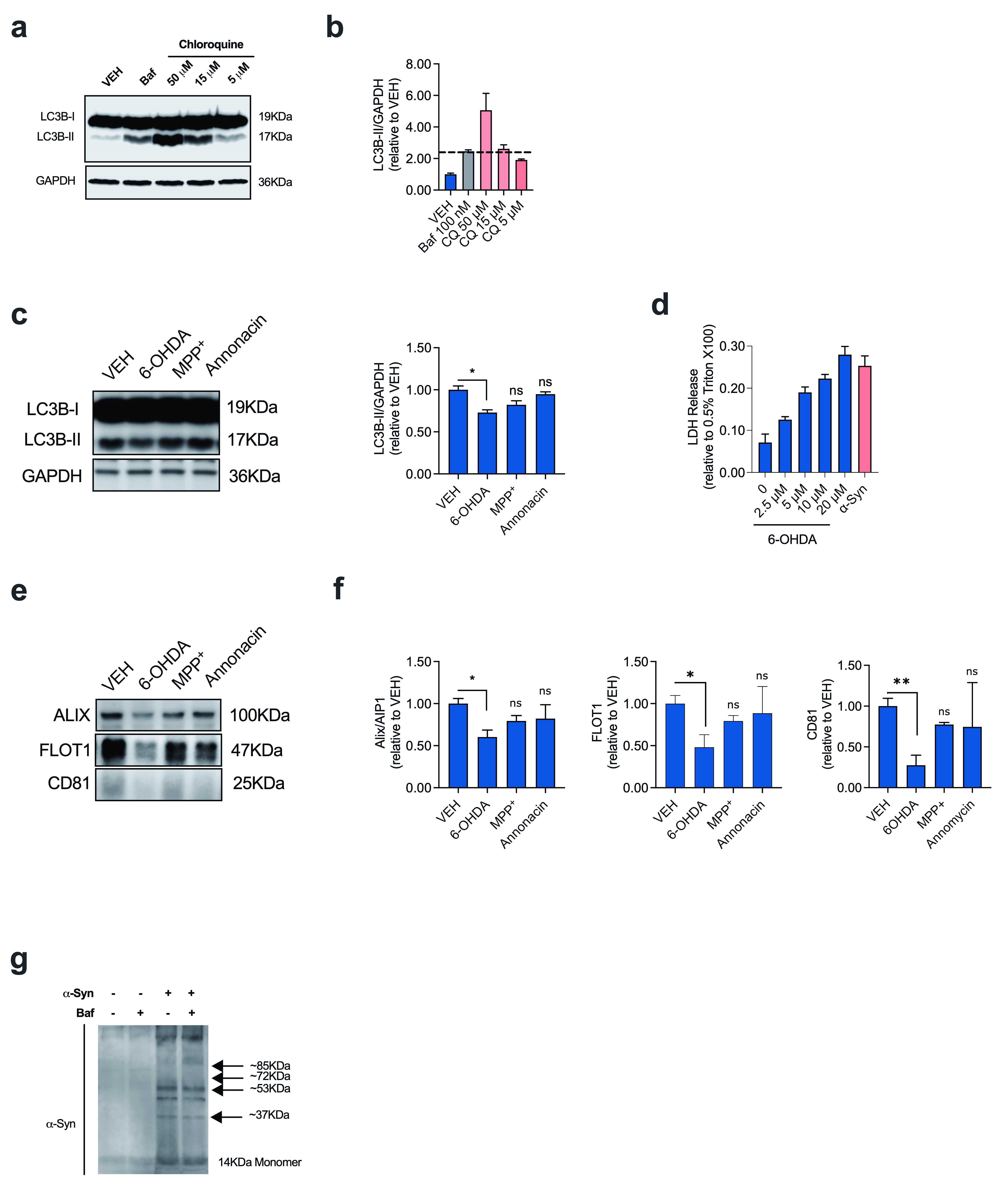
**

**Supplementary Figure 1 Neuronal cell death per se does not increase the abundance of LC3B-II or the abundance of EVs. a,b** Western blot and bar graph illustrating the effect of Chloroquine (CQ) on the abundance of LC3B-II. Treatment with CQ (24 hrs.) led to a dose-dependent increase in LC3B-II, similar to the autophagy inhibitor Bafilomycin A (Baf; 100 nM; 8 hrs) (n = 3/condition). **c** Western blot and bar graph illustrating the abundance of LC3B-I, -II and GAPDH in response to a 24 hrs. treatment of LUHMES cells with 6-OHDA (20 μM), MPP^+^ (10 μM) or annonacin (25 nM). 6-OHDA significantly decreased the abundance of LC3B-II, whereas MPP^+^ and annonacin had no significant effect (n = 3/condition). **d** Bar graph illustrating LDH release in response to treatment with increasing concentrations of 6-OHDA (for 24 hrs). The results indicate values relative to a positive control (cell lysis with 0.5% Triton X-100). Treatment with 20 μM 6-OHDA led to a similar degree of LDH release as the overexpression of α-Syn (n = 4/condition). **e,f** Western blot and bar graphs demonstrating that a 24 hrs treatment of LUHMES cells with 6-OHDA (20 μM), MPP^+^ (10 μM) or annonacin (25 nM) had either no effect on EV-associated proteins Alix/AIP1, Flotillin-1, and CD81 (MPP^+^, annonacin) or even decreased their abundance (6-OHDA) (n = 3/condition). **g** Western blot illustrating the abundance of monomeric and oligomeric α-Syn fractions in EV-enriched medium pellets from cultured LUHMES cells. Whereas Baf alone (100 nM, 8 hrs) had no effect, α-Syn overexpression increased the abundance of α-Syn oligomeric fractions in EVs (1^st^ and 2^nd^ lane from the right). For comparison of the means, a two-tailed unpaired t-test was used in panel c, f *P < 0.05. Data are shown as means ± SEM.

**Supplementary Figure 2:**

**
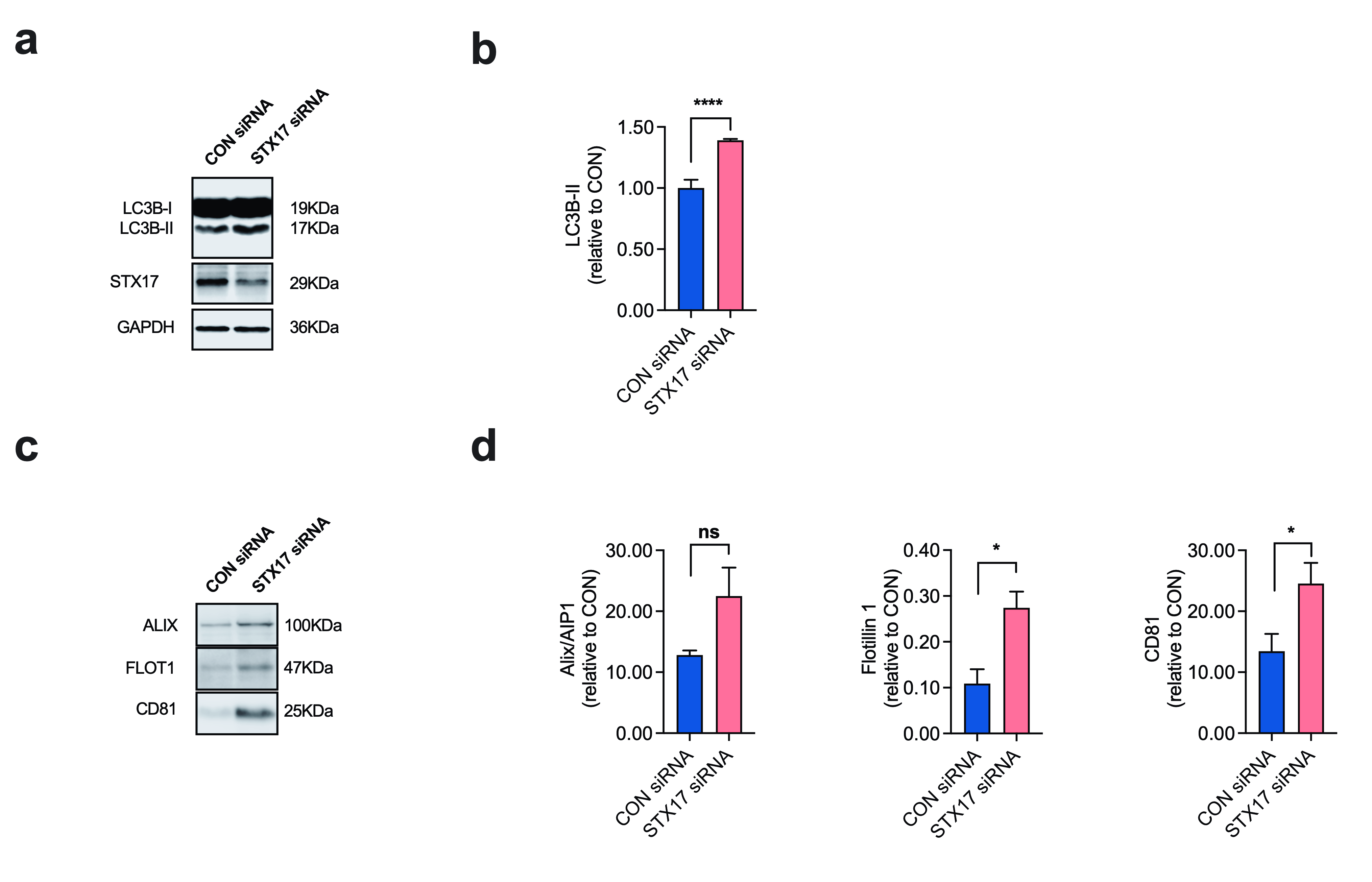
**

**Supplementary Figure 2: Knocking down STX17 mimics the effect of α-Syn on LC3B-II and EV release. a,b** Western blot and bar graph illustrating an increase of LC3B-II in response to transfection of cells with a STX17-specific siRNA (n = 9/condition). **c,d** Western blot and bar graph illustrating in increased abundance of the EV-associated proteins Alix/AIP1, Flotillin-1 and CD81 in EV-enriched medium pellets from STX17-siRNA-transduced cells compared to CON siRNA transfection (n = 4/condition). For comparison of the means, a two-tailed unpaired t-test was used in panels b,d; ***P < 0.005, *P < 0.05. Data are shown as means ± SEM.

**Supplementary Figure 3**

**a**

**b**

**c**

**
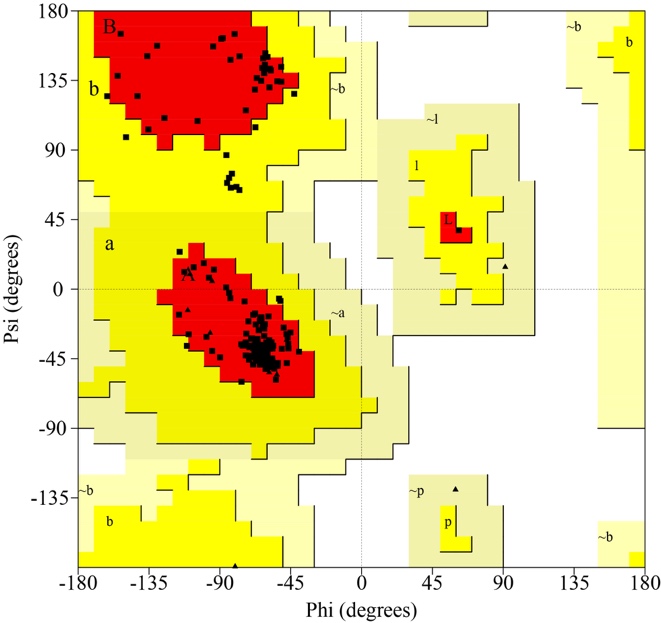
**

**
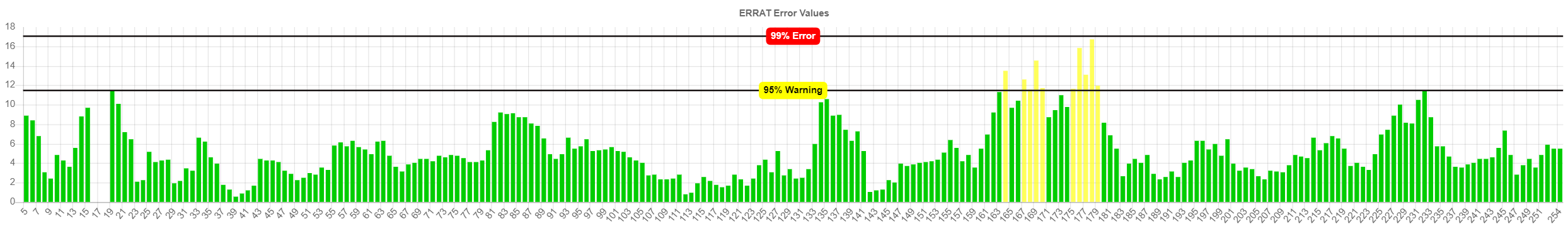

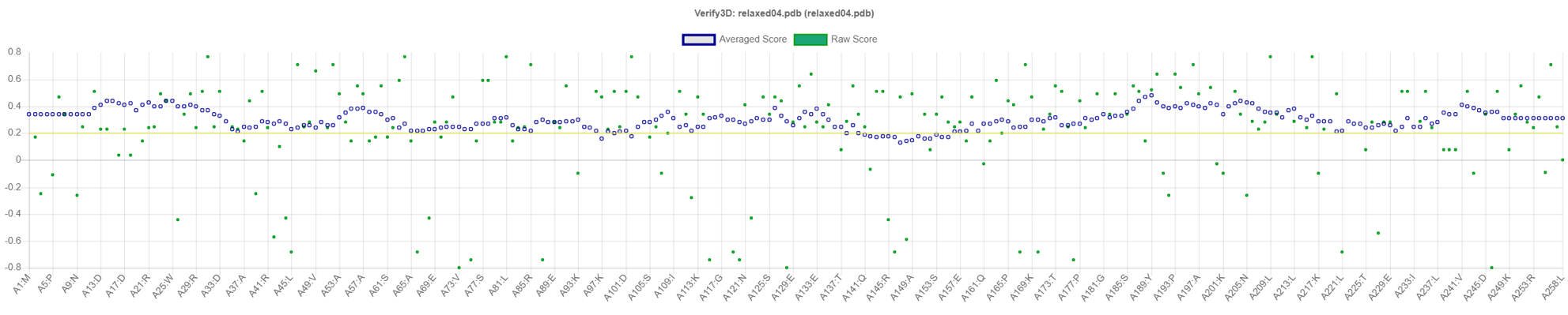
**

**
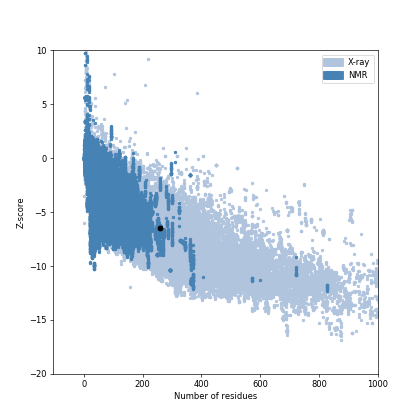
**

**d**

**Supplementary Figure 3 Validation results of the SNAP29 protein 3D structure model. a** Verify 3D plot showing our SNAP29 3D structure model has more than 80% (93.41%) of the residues scoring ≥ 0.2 in the 3D/1D profile **b** ERRAT plot demonstrating the overall quality of the predicted structure model. Regions that can be rejected at a 95% confidence level are highlighted in yellow. No region exceeds the 99% error value (red). **c** Ramachandran plot from PROCHECK analysis, showing most of the residues in the “favored” regions (red), few in the “allowed” regions (yellow), and no residue in the “generously allowed” regions (light yellow) or “disallowed” regions (white). **d** ProSA plot showing the z-score (highlighted as a black dot) of our model.

**Supplementary Figure 4:**


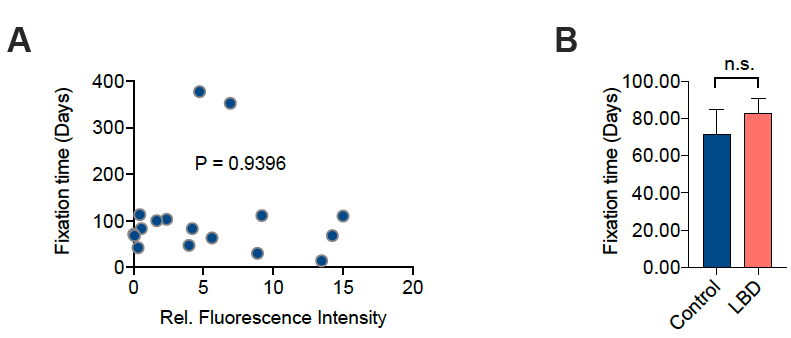


**Supplementary Figure 4** **Tissue fixation time has no effect on the intensity of the SNAP29 fluorescence signal**. **a** Scatter blot demonstrating no correlation of tissue fixation time and fluorescence intensity. Each blue circle represents an individual case. A two-tailed t-test has been used for analysis. **b** Bar graph illustrating no significant difference in the fixation time between control and LBD cases. For comparison of the means, a two-tailed unpaired t-test was used in panel b. Data are shown as means ± SEM.

**
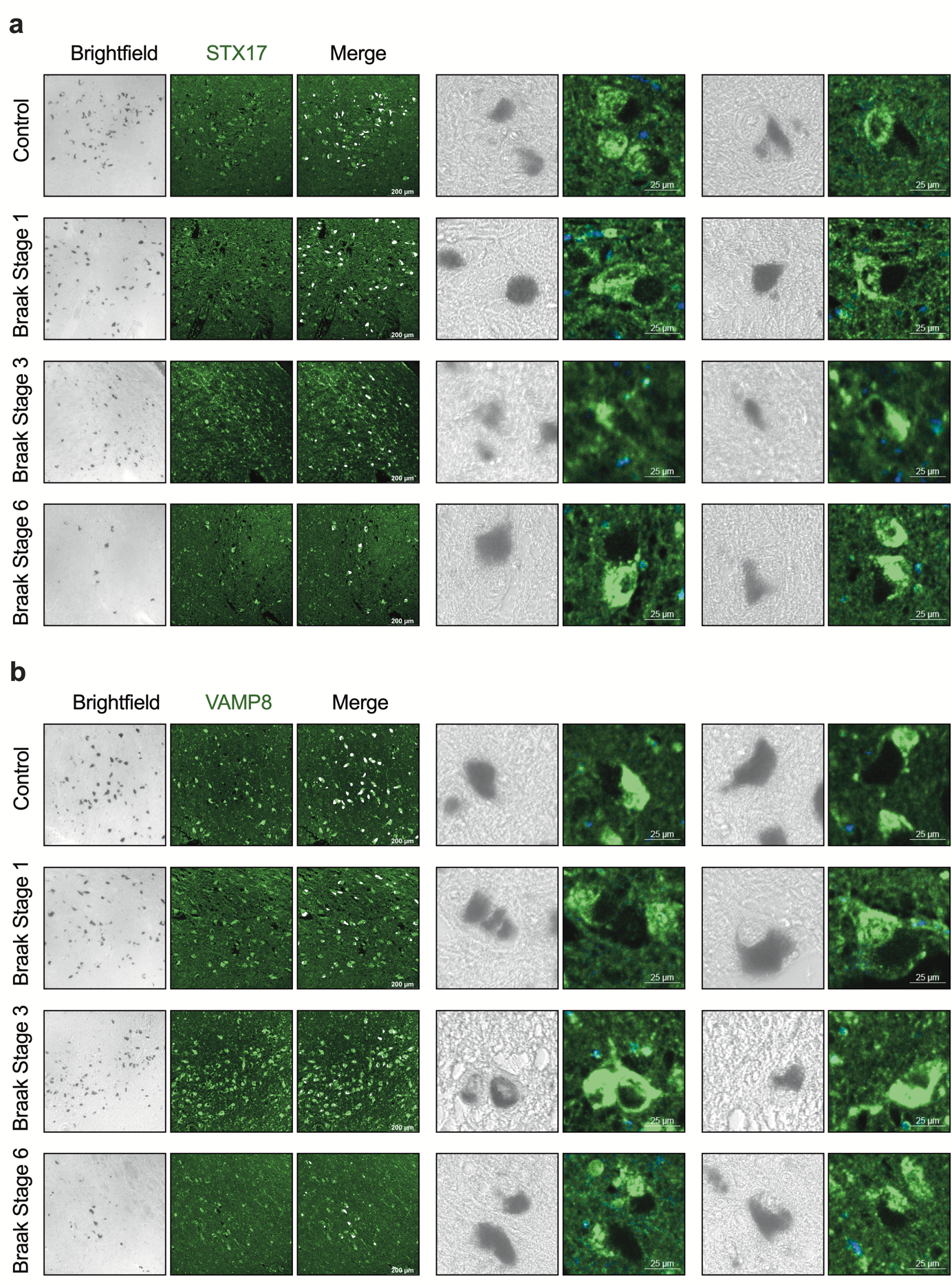
**

**Supplementary Figure 5** **STX17 and VAMP8 remain unchanged in SNc neurons during LBP. a,b** Representative photomicrograph from immunohistochemical staining of SNc post-mortem brain tissue at a low (bar: 200 μm) and high (bar: : 25 μm) magnification. Bright field images indicate neuromelanin pigment (black) in SNc DA neurons. Tissue sections were stained with antibodies against STX17 or VAMP8, revealing a preserved abundance of these proteins across all LBP stages.
