# Supplementary material for: Alpha-Synuclein defects autophagy by impairing SNAP29-mediated autophagosome-lysosome fusion
Source: Cell Death Dis. 2021 Sep 17;12(10):854. doi: 10.1038/s41419-021-04138-0 (PMC8448865; doi:10.1038/s41419-021-04138-0)
Supplement: Supplementary file 3 — Author Contribution Form [file 41419_2021_4138_MOESM3_ESM.pdf]

DECLARATION OF CONTRIBUTIONS TO ARTICLE

ADMC

Manuscript Number:	Journal Name:	
CDDIS-21-1344R	*Cell Death & Disease*	(the 'Journal')

Proposed Title of the Contribution:

Alpha-Synuclein defects autophagy by impairing SNAP29-mediated autophagosome-lysosome fusion (the 'Contribution)

Author(s):

Qilin Tang; Pan Gao; Thomas Arzberger; Matthias Höllerhage, Jochen Herms, Günter Höglinger & Thomas Koeglsperger (the 'Authors')

For all *CDDis* articles, each person named as an author in the published version must be able to show he or she has contributed substantially to the article.

Authorship credit should be based on 1) substantial contributions to conception and design, acquisition of data, or analysis and interpretation of data; 2) drafting the article or revising it critically for important intellectual content; and 3) final approval of the version to be published. Authors should meet conditions 1, 2 and 3.

Any person who cannot be shown to have made a substantial contribution to the article cannot be listed as an author in the final version. The name of any person who is deemed to have made a minor contribution can, however, appear in the Acknowledgments section of the article.

Please complete the table below to indicate the contributions of all named authors to the manuscript.

Author Full Name:	Specification of Contribution to the Manuscript:
Qilin Tang	Made substantial contributions to conception and design, acquisition of data, analysis and interpretation, drafting the article and revising, and to the final approval of the version to be published.
Pan Gao	Made substantial contributions to the acquisition of data, analysis and interpretation, revising the article, and to the final approval of the version to be published.
Thomas Arzberger	Made substantial contributions to conception and design, acquisition of data, analysis and interpretation, revising the article, and to the final approval of the version to be published.
Matthias Höllerhage	Made substantial contributions to the acquisition of data, analysis and interpretation, revising the article, and to the final approval of the version to be published.
Jochen Herms	Made substantial contributions to conception and design, analysis and interpretation, revising the article, and to the final approval of the version to be published.
Günter Höglinger	Made substantial contributions to conception and design, analysis and interpretation, revising the article, and to the final approval of the version to be published.
Thomas Koeglsperger	Made substantial contributions to conception and design,analysis and interpretation, drafting the article and revising, and to the final approval of the version to be published.

Please complete the table below to indicate the contributions of all named authors to the figures.

Figure 1:

QT, PG, MH, JH, GH, TK

Figure 2:

QT, PG, MH, JH, GH, TK

Figure 3:

QT, PG, MH, JH, GH, TK

Figure 4:

QT, PG, MH, JH, GH, TK

Figure 5:

QT, PG, MH, JH, GH, TK

Figure 6:

QT, PG, MH, JH, GH, TK

Fig. 7: QT, PG, TA, MH, JH, GH, TK
Fig. 8: QT, TK

Signed for and on behalf of the Author(s):	Print Name:	Date:
	Köglsperger	06/12/2021
